# Flexible methods for uncertainty estimation of digital PCR data

**DOI:** 10.1016/j.isci.2025.111772

**Published:** 2025-01-08

**Authors:** Yao Chen, Ward De Spiegelaere, Matthijs Vynck, Wim Trypsteen, David Gleerup, Jo Vandesompele, Olivier Thas

**Affiliations:** 1 Department of Applied Mathematics, Computer Science and Statistics, Ghent University, 9000 Ghent, Belgium; 2 Digital PCR Center (DIGPCR), Ghent University, 9000 Ghent, Belgium; 3 Department of Morphology, Medical Imaging, Orthopaedics, Physiotherapy and Nutrition, Ghent University, 9820 Merelbeke, Belgium; 4 Department of Internal Medicine, Ghent University and University Hospital, 9000 Ghent, Belgium; 5 OncoRNALab, Center for Medical Genetics, Department of Biomolecular Medicine, Ghent University, 9000 Ghent, Belgium; 6 Cancer Research Institute Ghent (CRIG), 9000 Ghent, Belgium; 7 pxlence, 9000 Ghent, Belgium; 8 Data Science Institute, I-BioStat, Hasselt University, 3590 Diepenbeek, Belgium; 9 National Institute for Applied Statistics Research Australia (NIASRA), University of Wollongong, Wollongong, NSW 2522, Australia

**Keywords:** Bioinformatics, Bioinformatic numerical analysis, Methodology in biological sciences

## Abstract

Digital PCR (dPCR) is an accurate technique for quantifying nucleic acids, but variance estimation remains a challenge due to violations of the assumptions underlying many existing methods. To address this, we propose two generic approaches, NonPVar and BinomVar, for calculating variance in dPCR data. These methods are evaluated using simulated and empirical data, incorporating common sources of variability. Unlike classical methods, our approaches are flexible and applicable to complex functions of partition counts like copy number variation (CNV), fractional abundance, and DNA integrity. An R Shiny app is provided to facilitate method selection and implementation. Our findings demonstrate that these methods improve accuracy and adaptability, offering robust tools for uncertainty estimation in dPCR experiments.

## Highlights

• Simulation shows binomial-assumption methods can inaccurately estimate standard error
• Two flexible methods improve standard error and confidence interval estimation for dPCR
• A free R Shiny app allows users to analyze dPCR data with a graphical interface

## Graphical abstract

**Figure:**
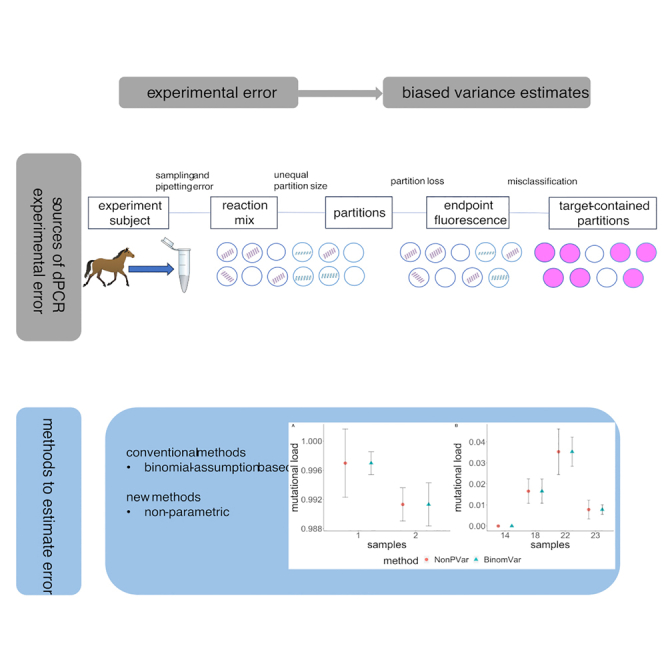

## Introduction

The use of a digital polymerase chain reaction (dPCR) to quantify nucleic acids has markedly increased in the last decade. The method involves massive partitioning of a sample in thousands of nanoliter-sized individual PCR reactors. dPCR has demonstrated many attractive characteristics, including a high accuracy, no need for a standard curve, and an unsurpassed repeatability.^1^^,^^2^^,^^3^ Thanks to this, dPCR is becoming the recommended method for highly precise quantification of nucleic acids, such as absolute concentration, minority species detection, copy number variation (CNV) estimation, fractional abundance quantification of mutations, linkage, template integrity, and many more.^4^^,^^5^^,^^6^^,^^7^^,^^8^ As a highly precise measurement method, dPCR theoretically offers enhanced repeatability (reduced variation within measurements conducted in the same experimental run) and reproducibility (diminished variation between measurements carried out in different experimental runs). These aspects are commonly assessed by examining the standard deviation of measurements.^9^^,^^10^^,^^11^^,^^12^

Accurate standard deviation estimation is crucial for enhancing the reliability of these methods, reducing false positives and negatives, and ultimately contributing to more robust scientific and clinical outcomes. For example, CNV is a critical factor in cancer research. Accurate estimation of CNV is essential for understanding disease mechanisms, developing targeted therapies, and making informed clinical decisions.^13^ Poor standard deviation estimates could lead to incorrect CNV calls, resulting in misinterpretation of genetic risk factors or therapeutic targets, potentially impacting patient outcomes.^13^ Similarly, fractional abundance measurements are crucial in liquid biopsy applications, where detecting and quantifying low-frequency mutations in circulating tumor DNA is essential for early cancer detection, monitoring treatment response, and identifying resistance mutations.^14^ In this context, a reliable standard deviation estimate of fractional abundance is critical to distinguish between true biological signals and technical noise, ensuring reliable clinical decisions. DNA integrity is an important quality control measure in various genomic applications, such as next-generation sequencing and forensic analysis.^15^ Accurate standard deviation estimation here is vital to assess the quality and reliability of DNA samples, influencing downstream analyses and interpretations.

In dPCR, calculations are based on a binary outcome: a partition can be either positive or negative, respectively indicating the presence of one or more target nucleic acids or its absence in a partition. Absolute quantification of the target nucleic acids is subsequently based on the Poisson distribution that estimates the average number of target molecules per partition. Because of the binary outcome of partition classification, a binomial distribution is assumed. This allows for the calculation of a theoretical measure of uncertainty, such as a standard deviation and a confidence interval (CI) for a single reaction.^16^ However, previous work has indicated that the assumption of a binomial distribution may not be valid in dPCR because additional sources of variation are not accounted for.^17^^,^^18^^,^^19^

The binomial or multinomial assumption for the number of positive partitions, imposed for single- and multiplex experiments, respectively, stands when there is only sampling variation present.^17^^,^^20^ However, this may not be realistic because other important sources of bias and variability may come in during the pre-analytical, analytical, and data analysis phase of dPCR experiments.^17^ For example, pipetting errors may be introduced when preparing the specimen, partition volume variation comes to play during the experiment,^17^^,^^21^ or misclassification of partitions may arise after the amplification and reaction readout process. Hence, the binomial or multinomial assumption will be violated and the existing methods will fail to provide correct results.

A popular method for estimating the variance of a non-linear function of counts is the delta method.^22^ The core idea behind the delta method is to simplify a nonlinear function by using a linear approximation. By doing this, we can more easily propagate the error from the input variable to the output of the function. A non-linear function of counts means that the quantity of interest is not linearly related to the counts, such as a ratio of counts or the logarithm of a count as in Poisson statistics for absolute quantification. The delta method approximates the non-linear function by a linear function of counts, whereby the variance estimator of a linear function is straightforward if the distribution of the count is known (e.g., a binomial distribution for the number of positive partitions). Logarithmic and exponential functions are often well approximated by the delta method, but ratios, such as CNVs, are often not well approximated by a linear function and hence the delta method may perform poorly. Moreover, for every quantity of interest some mathematical operations (e.g., differentiation of the nonlinear function) are required, which may be cumbersome.^23^

This paper focuses on generic methods for uncertainty estimation, more precisely variance and CI estimation for all quantities of interest (absolute quantity, CNV, fractional abundance, …), without requiring mathematical operations. We propose two methods, named BinomVar and NonPVar.

## Results

### Simulation results

For absolute quantification, the estimators of λ were nearly unbiased (relative bias ⟨0.5%), except in the presence of partition size variation and misclassification (Figures S2–S7). The effect of partition size variation was limited (the relative bias can increase to 1%), but misclassification had a larger impact with relative bias as large as 10%; this agrees with the findings of the study by Jacobs et al.^17^

**Figure 1:**
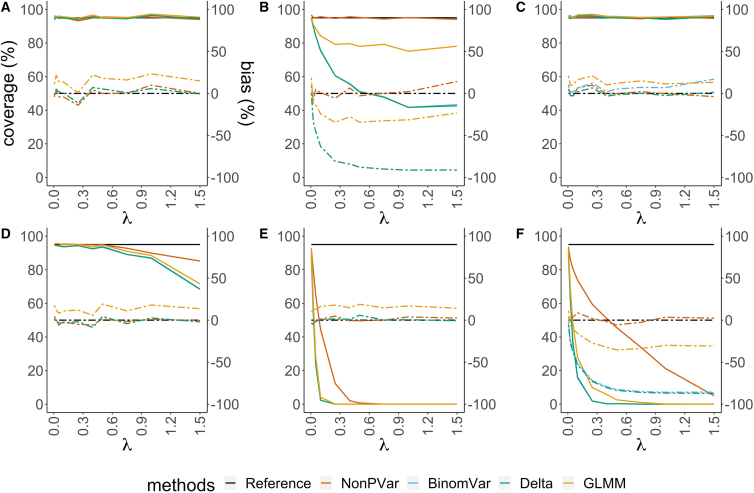
Empirical coverage of the 95% CIs (solid lines, left axis) and relative bias of the variance estimates (dashed lines, right axis) for absolute quantification in different scenarios (A–F) The horizontal axes represent varying concentrations of target molecules from low to high. (A) only sampling variation and random partitioning (B) 3% pipetting error (C) 20% partition loss (D) coefficient of variation of 10% in partition size (E) misclassification with 0.01% false positive rate and 5% false negative rate (F) all variation included. The reference for empirical coverage is set at 95% (black solid line). The constructed CIs are expected to cover the true values in 95% of the cases. The closer other solid lines are to this reference, the better the CIs are. The reference for relative bias is set at 0% (black dashed line). The closer other dashed lines are to this reference, the lower the relative bias is.

With only sampling variability and random partitioning, all methods gave good CIs (empirical coverage ≈95%, Figure 1). In the presence of pipetting error, both the delta method and BinomVar covered the true value with a probability of less than 50% when λ>0.5 (Figure 1B). The NonPVar method was more robust against such errors and performed best. The GLMM method was the runner-up.

In terms of the variance estimation, NonPVar had low relative bias in all scenarios (the absolute value of the relative bias ⟨5%). With additional pipetting error, the NonPVar variance estimator was much less biased compared to BinomVar, delta method, and GLMM. The absolute bias and the distribution of the variance estimates were also checked (Figures S8–S19). In particular, the results (boxplots of the variance estimates) show that the NonPVar estimates had a larger variance than the alternative methods. Consequently, the variance estimates were less precise and less stable compared to the other methods. This is due to the empirical nature of the NonPVar method for estimating the variance (in the absence of distributional assumptions), which typically only uses a few replicates. With additional pipetting error, only variance estimates by NonPVar and GLMM were close to the true value. The BinomVar and delta methods underestimated the variance. Partition size variation and especially misclassification had an impact on the CI coverage. All methods failed to cover the true value when partitions were misclassified and the error was consistent for all replicates.

For CNV in singleplex, target and reference molecules are quantified separately. This means that additional sources of variability, such as pipetting errors will be different for target and reference molecules. In this case, pipetting errors can’t cancel out, with a concomitant impact on the variance estimation (see Figure S20). The effects of unequal partition size and misclassification were not negligible. NonPVar performed at least as good as the other methods in terms of empirical coverage, while its relative bias remained quite low (the absolute value of the relative bias ⟨5%.

**Figure 2:**
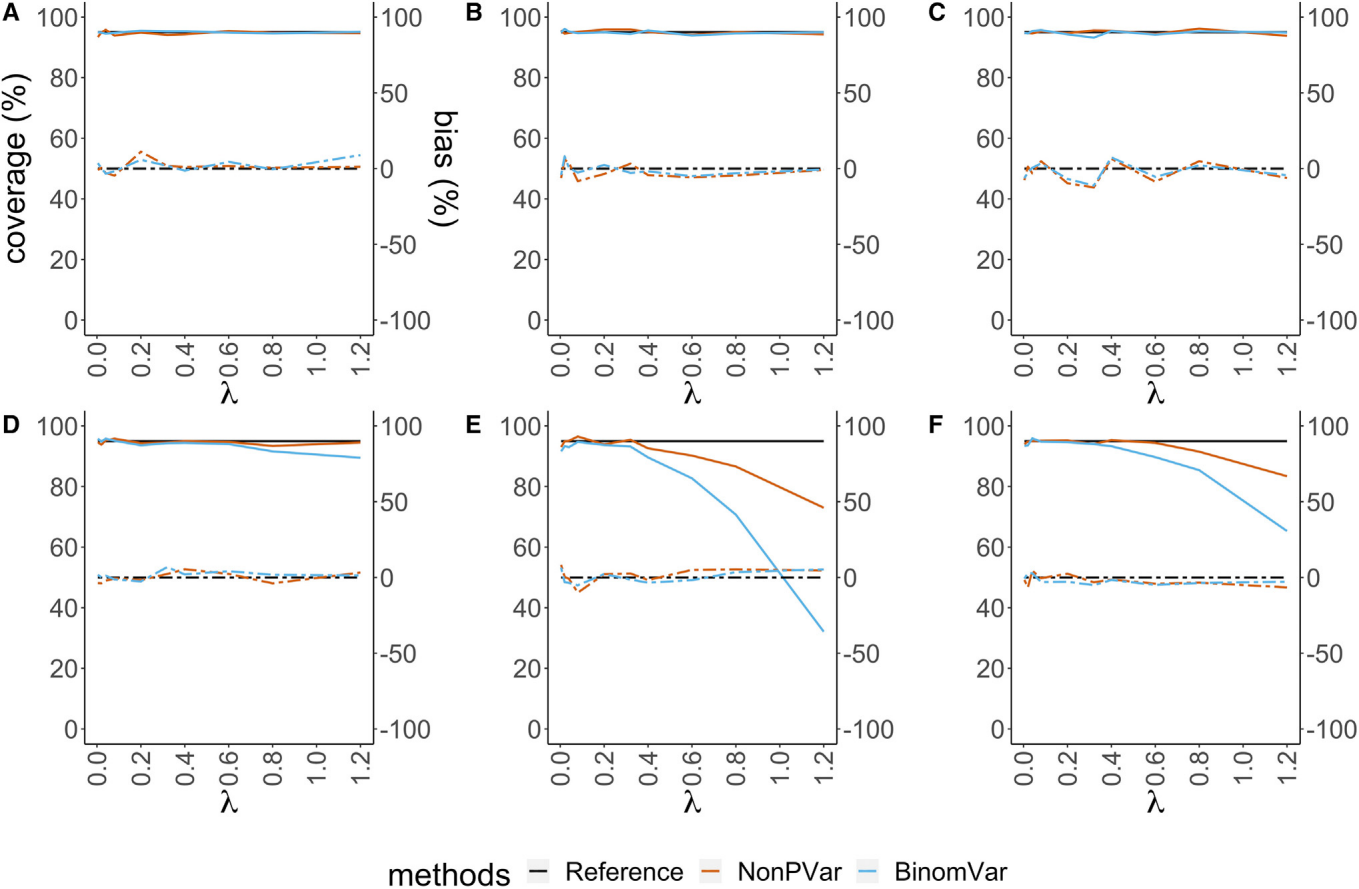
Empirical coverage of the 95% CIs (solid lines, left axis) and relative bias of the variance estimates (dashed lines, right axis) for high DNA integrity (=80%, that is, 20% of the target molecules are fragmented) in different scenarios (A–F) The horizontal axes represent varying concentrations of intact molecules from low to high. (A) only sampling variation and random partitioning (B) 3% pipetting error (C) 20% partition loss (D) coefficient of variation of 10% in partition size (E) misclassification with 0.01% false positive rate and 5% false negative rate (F) all variation included. The reference for empirical coverage is set at 95% (black solid line). The constructed CIs are expected to cover the true values in 95% of the cases. The closer other solid lines are to this reference, the better the CIs are. The reference for relative bias is set at 0% (black dashed line). The closer other dashed lines are to this reference, the lower the relative bias is.

In the simulations for DNA integrity, the concentration and intactness percentage were varied from low to high. Results in Figure 2 (see also Figures S61 and S62) show that the effect of pipetting error, which commonly had a big impact on the variation and CI of the estimates in absolute quantification or the CNV singleplex set-up, canceled out. The empirical coverage of BinomVar was close to 95%, even in the presence of pipetting error. The relative biases of NonPVar and BinomVar were both close to 0. However, the NonPVar estimates were less precise (Figures S49–S60), as also observed for absolute quantification (see earlier).

For fractional abundance of a mutation, results in Figure S40 show that without misclassification, the performances of NonPVar and BinomVar were quite comparable. With misclassified partitions, the empirical coverage of BinomVar was similar to that of NonPVar in low or medium concentration scenarios, but it was considerably lower in the high concentration scenarios.

In CNV duplex, all target DNA molecules are quantified within the same reaction, and thus additional sources of variation apply equally to them. The pipetting error is canceled out and the effect of partition size variation diminished, as in DNA integrity and fractional abundance of a mutation (see Figure S39). The variance estimates are still accurate despite the errors.

### Case study

For the mutation data, CIs given by BinomVar and NonPVar were quite different for some samples while for others they were comparable. Note that the sample concentrations were low, and that the simulation results show that at low concentration, the random sampling variability was dominating. Other sources of error, such as misclassification, did not have a big impact. In this scenario, BinomVar was expected to give more precise variance estimates and thus should be preferred.

**Figure 3:**
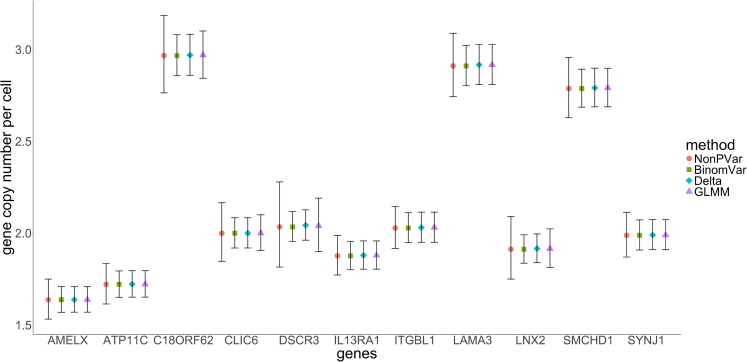
CIs of estimated copy numbers in sample 15 after normalization using the CLIC6 locus (accounting for inter-replicate variability) This is an example of what the output of the web app looks like. Data are represented as mean ± SEM.

For the CNV dataset, CIs given by BinomVar, the delta method and GLMM were overall close (Figures 3 and S63 for more details). It is important to observe that for the gene *DSCR3*, the NonPVar approach produced a CI that was over twice as wide as those generated by BinomVar and the delta method. This significant disparity may suggest the possibility of additional error sources beyond sampling variability contributing to the large variance among replicates. In this case, the binomial assumption was likely too optimistic.

### Demonstration of the R shiny app

**Figure 4:**
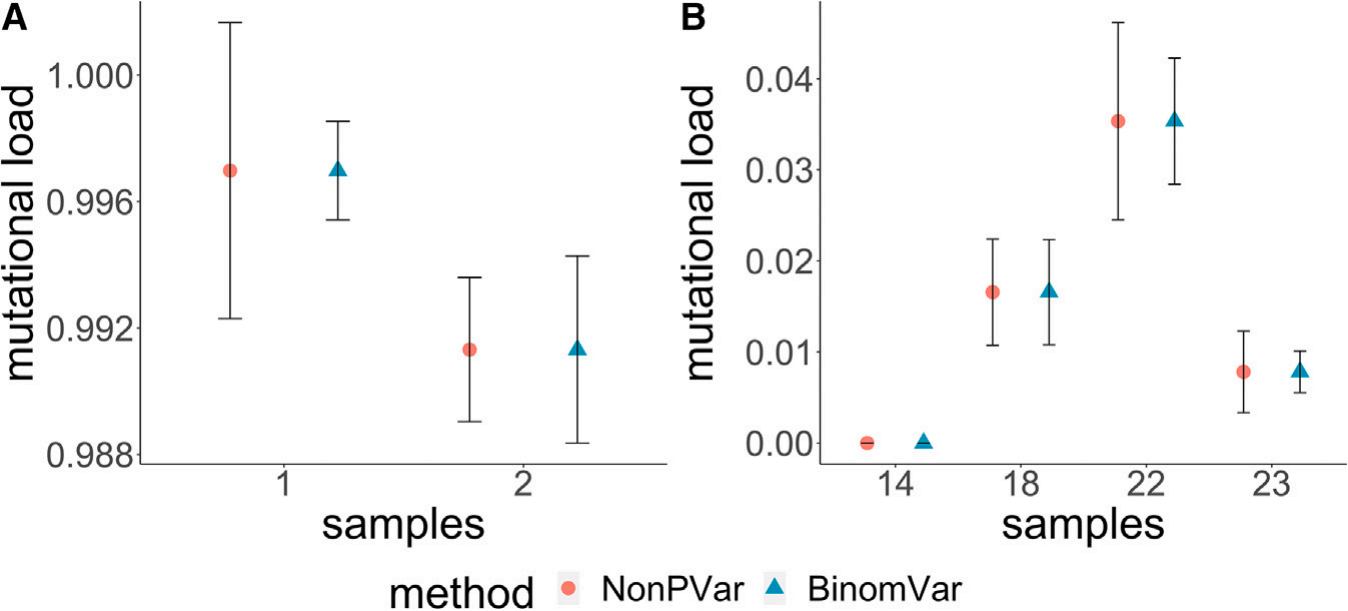
The plot shows CIs of the fractional abundance of a mutation calculated with BinomVar and NonPVar from the mutation data Data are represented as mean ± SEM. (A) Samples 1 and 2. (B) Samples 14, 18, 22, and 23.

An R Shiny app was developed to enable estimation and visualization of the CIs. Here, sample 1 and 2 of the fractional abundance data (see Figure 4) are used for demonstration.

**Figure 5:**
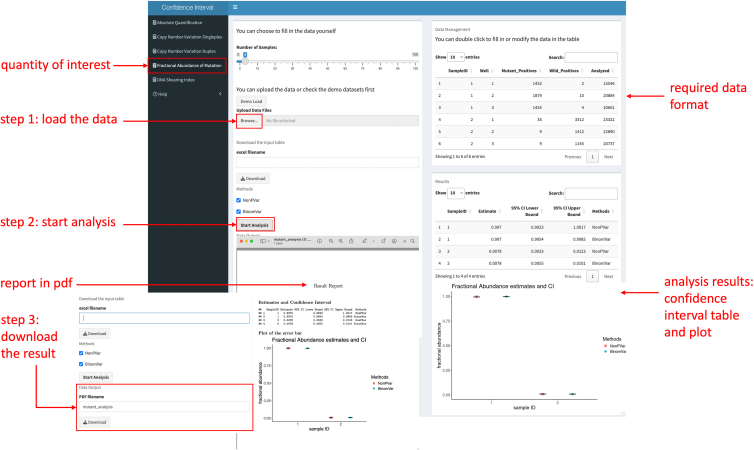
Illustration of the Shiny app with an example of fractional abundance estimation This figure shows the various steps involved in using the web app, including selecting the quantity of interest, loading the data, starting the analysis, and downloading the results. The right panel presents the analysis results, including a table of confidence intervals and a plot of fractional abundance estimates with confidence intervals.

As a first step, the quantity of interest must be selected. The next step is to load the data into the application; the appropriate data format can be learned from the demo data that come with the Shiny app. Following the data loading step, users can initiate the analysis by clicking the “Start Analysis” button. The Shiny app then processes the data, and within seconds generates a comprehensive output, comprising both a table and a figure showing the CIs. A download option is provided to facilitate further exploration. See Figure 5 for a visualization of the process.

## Discussion

In digital PCR data, variation stems not only from random partitioning and sampling, but also from additional sources like pipetting errors, partition misclassification, partition size variability, etc. Traditional methods such as the delta method for estimating uncertainty of the quantities of interest do not consider those additional experimental sources of variability.

These different sources of variability have different impacts on the variance estimates (Figures 1, 2, S20, S39, and S40). In absolute quantification and the CNV singleplex set-up, ignoring pipetting error will result in an underestimation of the variance and in an inaccurate CI. For CNV duplex, fractional abundance of a mutation or DNA integrity, most of the additional errors cancel out. The effect of varying partition size does not disappear even in duplex or multiplex set-ups, because all methods make use of the Poisson assumption for calculating $\hat{\lambda }$. This assumption is violated if the partition volume is not constant. The impact of misclassification does not cancel out either. This indicates a clear need to use a good partition classification method.

The use of traditional methods may thus lead to underestimation of the variance and hence to too narrow CIs.^17^ Furthermore, other quantities of interest such as fractional abundances of mutations and DNA integrity are relevant outcomes in dPCR, but their variance estimations are challenging. To address these challenges, we have introduced two methods, BinomVar and NonPvar.

The BinomVar method gives more precise estimates, because it relies on the Poisson assumption for the sampling distribution of the number of molecules over the replicates (that is, binomial assumption for the number of positives). The price that BinomVar pays is that it becomes less robust when this distributional assumption is violated. NonPVar is a data-driven method and does not rely on a distributional assumption for the number of positive partitions; the variance is inferred from the data. The method is more robust in the presence of additional sources of error, such as pipetting errors. Since the method makes use of replicates, the estimation accuracy depends on the number of replicates. This method, despite being unbiased for many scenarios, requires a sufficient number of replicates to give good variance estimates. Without an adequate number of samples, the variance estimates may be highly sensitive to small changes in the data, leading to increased uncertainty and potentially unreliable results. The sample size can be calculated based on the required precision^24^ and power.^25^ Note that the CIs provided by both methods are based on the assumption of the asymptotic normality of the estimator λ. According to the central limit theorem (CLT), for a sufficiently large sample size, the distribution of the sample mean approaches a normal distribution, regardless of the original distribution of the data.^22^ However, this normal approximation may not fully capture the true uncertainty when the sample sizes are small, where deviations from normality can be more pronounced.

The results also show that at low concentration scenarios, other sources of errors, such as pipetting error, do not have a big impact and BinomVar is the better choice. When the delta method is available (that is, when the mathematical derivation and the resulting formula for the variance are already established), it gives similar results as BinomVar. In this case, we would recommend the delta method because it is faster than the computationally intensive bootstrap. In summary, we would suggest choosing estimation methods by the type of experiments, concentration levels, and number of replicates; see Table 1.

**Table 1:** Recommended variance estimation methods

concentration	type of experiment	recommended method(s)
low	singleplex	BinomVar, GLMM or Delta method
	duplex/multiplex	BinomVar, GLMM or Delta method
high	singleplex	NonPVar
	duplex/multiplex	Depends on the classification. If the clusters are well-separated, then BinomVar, GLMM or Delta method. Otherwise, misclassification error will be high and NonPVar is a better choice.

The choice of method also depends on the sample size. If the precision requirement is met, NonPVar will be as good as other methods in duplex/multiplex scenarios. Note there is no exact threshold to define low or high concentration levels. In our simulation studies, λ<0.1 was considered as low concentration level. However, it also depends on the quality of the data. If there is low pipetting error and targets are accurately quantified, then the threshold should be higher.

When multiple methods are available for a given scenario, users can choose based on ease of use and computational requirements. BinomVar is the easiest to use, followed by the GLMM, and then the delta method. However, in terms of computational demand, BinomVar is the most intensive, followed by GLMM, with the delta method being the least computationally demanding.

It is important to note that the delta method remains a valid approach for absolute quantification and CNV. However, for other quantities of interest, deriving mathematical formulas for variance estimation using the delta method can be challenging. When the function is very nonlinear, the relationship between the input variables and the output of the function cannot be well depicted by a line, such as in the case of ratios. In such cases, the delta method will work less well and BinomVar may be a better choice.

It is recommended that users apply all the available methods—NonPVar, GLMM, BinomVar, and the delta method—to estimate variance. A significant difference in the results between these methods may indicate the presence of unexpected or unknown additional sources of error in the measurements.

### Limitations of the study

Our study introduces two methods for variance estimation across various quantities of interest. We provide recommendations for method selection based on the type of experiment and concentration levels; however, the choice of method also depends on sample size. In this study, we do not define specific sample size criteria. NonPVar performs comparably to other methods as the number of replicates increases, which influences method selection. Future work should further explore the interplay between sample size, concentration levels, and experimental types. Additionally, for CNV data, multiple reference genes are often available. Both BinomVar and NonPVar can estimate CNV using each reference gene, but an approach to integrate estimates from different references could yield a more stable and accurate CNV estimate, as done in the study by Vynck et al.^26^

## Resource availability

### Lead contact

Further information and requests should be directed to and will be fulfilled by the lead contact Olivier Thas (olivier.thas@uhasselt.be).

### Materials availability

This study did not generate new unique reagents.

### Data and code availability

• Data: this article analyzes existing, publicly available data. The details are listed in the key resources table.
• Code: our source code is available on GitHub (https://github.ugent.be/DIGPCR/VarianceEstimate) and an R shiny web application is also available at https://digpcr.shinyapps.io/variance_estimate/.
• All other requests: Any additional information required to reanalyze the data reported will be shared by the lead contact upon request.

## Acknowledgments

This work was funded by the Ghent University Special Research Fund, 10.13039/501100007229BOF (grant 01IO0420).

## Author contributions

Conceptualization: Y.C. and O.T.; implementation: Y.C.; supervision: O.T.; original draft: Y.C.; review and editing: Y.C., W.D.S., M.V., W.T., D.G., J.V., and O.T.

## Declaration of interests

M.V. is supported by funding provided by Stilla Technologies. J.V. is co-founder of pxlence.

## STAR★Methods

### Key resources table

**Table:** 

REAGENT or RESOURCE	SOURCE	IDENTIFIER
**Biological samples**		

CNV data	Vynck et al.^26^	https://doi.org/10.1016/j.bdq.2016.06.001
Mutation data	Tytgat et al.^8^	https://doi.org/10.1093/clinchem/hvab021

**Software and algorithms**		

Variance estimation codes	This paper	https://github.ugent.be/DIGPCR/VarianceEstimate
Variance estimation R shiny app	This paper	https://digpcr.shinyapps.io/variance_estimate/

### Experimental model and study participant details

The datasets used in this study were obtained from previously published papers and are publicly available. Due to ethical and privacy restrictions, detailed information about most samples, such as age and gender, is not provided. However, we do not expect these factors to have any impact on the results.

For the CNV data, DNA was extracted from blood samples of two individuals with chromosomal abnormalities using the QIAamp DNA Blood Mini Kit (Qiagen) following the manufacturer's protocol. 14 genes of interest were analyzed, comprising 13 target loci located on chromosomes 13, 18, 21, X, and Y, along with a single reference locus (RPP30) on chromosome arm 10q used for normalization.

For the mutation data, the patient samples were obtained from a female donor around 30 years old with a high mutational load at the m.11778 locus. Following ovarian stimulation, multiple cumulus oocyte complexes were retrieved, including metaphase II (MII) oocytes, one metaphase I (MI) oocyte, and one germinal vesicle (GV) oocyte. Additional samples included a non-patient enucleated MII oocyte (sample 14), two patient zygotes (samples 8 and 9), and two non-patient *in vitro*-matured (IVM) oocytes (samples 15 and 16).

### Method details

#### Poisson statistics

A typical dPCR data analysis starts from the end-point fluorescence after a fixed number of amplification cycles. The raw continuous fluorescence levels are transformed to binary (digital) observations after applying a threshold. In particular, when the end-point fluorescence exceeds the threshold, the partition is labelled positive, otherwise negative. Let n denote the number of partitions. The relation between the binary outcome $Y_{j}$ of partition j and the unobserved count of the target molecule $Y_{j}^{*}$ in that partition can be formulated as j=1,…,n

(Equation 1) $$Y_{j}=\min\left(Y_{j}^{*},1\right)=\left\{ \begin{array}{cc} 0 & \;\text{if}\;Y_{j}^{*}=0\\ 1 & \;\text{otherwise}\; \end{array}\right.,$$

i.e. $Y_{j}$ is 0 if there are no copies and it is 1 if there is at least one copy.

As the total number of molecules (m) in the sample is fixed and the entry of a molecule into a partition is random, the counts $Y_{j}^{*}$ follow a binomial distribution with distribution function

(Equation 2) $$P\left(Y_{j}^{*}=y|m,n\right)=\left(\begin{array}{c} m\\ y \end{array}\right){\left(\frac{1}{n}\right)}^{y}{\left(1-\frac{1}{n}\right)}^{m-y}\text{.}$$

When *n* is large enough, this binomial distribution can be approximated by a Poisson distribution with parameter λ=m/n which can be interpreted as the average number of target molecules per partition. The distribution function of this Poisson distribution is given by

(Equation 3) $$P\left(Y_{j}^{*}=y|\lambda \right)=\frac{\lambda^{y}e^{-\lambda }}{y!}.$$

The λ parameter can be directly estimated from the digital outcomes, because $Y_{j}^{*}=0$ if and only if $Y_{j}=0$ (i.e. a partition containing no molecules is a negative partition). Upon using this relationship the Poisson distribution gives

(Equation 4) $$P\left\{Y_{j}^{*}=0|\lambda \right\}=\frac{\lambda^{0}}{0!}\exp\left(-\lambda \right)=\exp\left(-\lambda \right).$$

Hence the relationship (Throughout the article, log means natural logarithm.)

(Equation 5) $$\lambda =-\text{logP}\left\{Y_{j}^{*}=0|\lambda \right\}=-\text{logP}\left\{Y_{j}=0|\lambda \right\}.$$

Since the digital outcomes $Y_{j}$ are observed, the probability of a negative partition, $P\left\{Y_{j}=0|m,n\right\}$, can be estimated by 1−K/n, where $K=\sum_{j=1}^{n}I\left(Y_{j}=1\right)$ is the number of positive partitions. The estimate of λ thus becomes

(Equation 6) $$\hat{\lambda }=-\log\left(1-\frac{K}{n}\right).$$

This parameter estimate is crucial in most of the dPCR applications. For example, in absolute quantification the concentration of the target is estimated as $\hat{\lambda }/V_{p}$, with $V_{p}$ the (average) volume of a partition. Another example: CNV is based on the ratio of two $\hat{\lambda }$’s; e.g. one for the target and one for the reference.

The imprecision of the estimate $\hat{\lambda }$ can be expressed as its standard error $\text{se}\left(\hat{\lambda }|m,n\right)$ or its variance $\text{Var}\left\{\hat{\lambda }|m,n\right\}={\text{se}}^{2}\left(\hat{\lambda }|m,n\right)$. The focus of this paper is on the estimation of this variance. Imprecision can also be expressed as a confidence interval (CI) of λ. If the sampling distribution of $\hat{\lambda }$ is approximately normal, then an approximate 95% CI can be calculated as $\hat{\lambda }\pm 1.96\times \hat{\text{se}}$, with $\hat{\text{se}}$ the estimated standard error. Later we will also propose another method for CI calculations. Since $\hat{\lambda }$ is a function of the number of positives K, the variance of $\hat{\lambda }$ depends on the distribution of K.

#### Existing methods

The conventional method for estimating the variance of the Poisson parameter ($\hat{\lambda }$) employs the delta method.^27^^,^^28^ In this approach, first, the variance of the count of positive partitions (k) is estimated based on the binomial/multinomial distribution. Subsequently, the variance of $\hat{\lambda }$, which is a function of k, is approximated with the delta method. The delta method has also been used for calculating the variance of CNVs.^27^^,^^28^ However, the conventional approach relies on the binomial/multinomial assumption for the count of positive partitions, which may be violated in practice.

Alternative methods for estimating variances and CIs for CNVs have been proposed. A numerical approach is introduced in^27^. The method builds on independent sampling distributions of the estimators for the reference and target. The distribution of CNVs is then approximated by all combinations of reference and targets, and accordingly, the 95% CI is established. Alternatively,^26^ used a generalized linear mixed model (GLMM) to derive CIs. This method allows for additional sources of variability, e.g., between-replicate variability, but it cannot be used for all types of quantities of interest. Only absolute quantification and CNV determination are included in this framework.

Indeed, to our best knowledge, the above methods have primarily been applied to absolute quantification and CNV estimation. However, there are other quantities of interest such as fractional abundances of mutations and DNA integrity (see further).

#### Quantities of interest

In this section a few examples of quantities of interest are given.

For absolute quantification, the Poisson parameter $\lambda_{i}$ (the average number of target molecules per partition in replicate i) is estimated as

(Equation 7) $${\hat{\lambda }}_{i}=-\log\left(1-\frac{k_{i}}{n_{i}}\right).$$

with $k_{i}$ the number of positive partitions, and $n_{i}$ the total number of partitions (Figure S1A). It is a non-linear function of $k_{i}$, but it can be well approximated by a linear function, which explains why the delta method works well here.

The estimate of CNV, on the other hand, is a ratio of Poisson parameters:

(Equation 8) $$\hat{{\text{CNV}}_{ij}}=\frac{\log\left(1-\frac{k_{Ai}}{n_{i}}\right)}{\log\left(1-\frac{k_{Bj}}{n_{j}}\right)}=\frac{{\hat{\lambda }}_{i}}{{\hat{\lambda }}_{j}},$$

with $k_{Ai}$ ($k_{Bj}$) and $n_{i}$ ($n_{j}$) referring to the number of positive partitions and total number of partitions for target molecule A (B) in replicate i (j) (in two separate singleplex reactions) and ${\hat{\lambda }}_{i}=-\log\left(1-k_{Ai}/n_{i}\right)$ (Figure S1B). This is also an example of a nonlinear function of the counts ($k_{Ai}$ and $k_{Bj}$). The linearization can be effective if the function does not strongly deviate from linearity within an interval where the observed counts are expected. However, in the case of ratios (particularly when the denominator is close to zero or highly variable), the range over which a linear approximation is valid can be quite limited. This difficulty in linearizing ratios makes it challenging to accurately model or analyze the relationship between the counts.

The fractional abundance of a mutation quantifies the proportion of the mutant alleles to the total amount of wild and mutant alleles (Figure S1B). The estimator for replicate i is given by,

(Equation 9) $${\hat{F}}_{i}=\frac{{\hat{\lambda }}_{Ai}}{{\hat{\lambda }}_{Ai}+{\hat{\lambda }}_{Bi}}.$$

with ${\hat{\lambda }}_{Ai}$ and ${\hat{\lambda }}_{Bi}$ the estimates of the Poisson parameter of mutant (A) and wild type (B) DNA, respectively in replicate i (typically in a duplex reaction).

Sequence linkage assays can be used to quantify DNA integrity, transgene quality control, linkage disequilibrium assessment or sequence inversions.^29^^,^^30^ These methods investigate a proportion and measure how many DNA fragments contain physically linked target sequences (Figure S1C).^7^^,^^31^ provided a method for estimating sequence linkage for the purpose of DNA integrity estimation, defining the integrity measurement as a ratio that reflects the proportion of intact DNA fragments. For replicate i,

(Equation 10) $${\hat{\text{Integrity}}}_{i}=1-\frac{\left({\hat{\lambda }}_{Ai}+{\hat{\lambda }}_{Bi}\right)/2}{\left(\left({\hat{\lambda }}_{Ai}+{\hat{\lambda }}_{Bi}\right)/2\right)+{\hat{\lambda }}_{ABi}}.$$

where $A_{i}$ and $B_{i}$ represent the estimated concentrations of broken (unlinked, single-positive) partitions for target sequences A and B, respectively and $AB_{i}$ represents the concentration of intact (double positive) partitions. This integrity measure is a ratio, where a value closer to 1 indicates a higher proportion of intact DNA fragments.

#### Proposed methods: general ideas

In a dPCR reaction, molecules undergo random partitioning, which introduces variation. This variation is the inherent variability of the partitioning process when there is no replicate and the number of molecules is fixed (see Section probabilistic framework in Section proposed methods: detailed description below for the distribution of the number of positives). When r replicates are available, we need to account for yet another level of variability: the numbers of molecules loaded in r replicated dPCR reactions show sampling variability, which is caused by sampling from a specimen (a larger volume). Even though the number of target molecules in a given specimen is fixed, the number of target molecules loaded onto the dPCR device will vary from replicate to replicate. Hence the number of loaded target molecules is considered a random variable, which is denoted by M (with $M_{i}$ is the number of molecules in the i− th replicate). The variance of $\hat{\lambda }$ is thus composed of two levels of variability: (1) the random sampling of M, and (2), given M, the random partitioning process. The details of the de-convolution into the sampling and random partitioning process can be found in Section probabilistic framework.

##### BinomVar: Binomial bootstrap for variances

Upon assuming that the numbers of molecules M are distributed over the replicates as a Poisson distribution, the distribution of the number of positives k is approximated by a binomial distribution (see Section probabilistic framework for a proof). In replicate i=1,…,r, this binomial distribution has parameters $n_{i}$ (the total number of partitions) and $\pi_{i}=1-e^{-\mu /n_{i}}$ the probability that a partition is positive. The parameter μ is the average number of molecules (averaged over replicates).

To circumvent the complex derivation of a mathematical formula for the variance, we propose a parametric bootstrap method by resampling numbers of positive partitions from this binomial distribution with the unknown parameter μ replaced with its estimate $\frac{1}{r}\sum_{i=1}^{r}{\hat{\lambda }}_{i}$. This bootstrap method will be referred to as BinomVar. Details of the algorithm are in Section binomial bootstrap process. It is important to note that the results of the delta method and BinomVar can be very similar, as both methods assume a binomial distribution for the number of positives. The key difference is that the delta method relies on mathematical derivations, whereas BinomVar circumvents this by using a bootstrap procedure. Unlike the delta method, BinomVar does not rely on a linearization of the QOI, which will work less well when the function is very nonlinear.

Based on the estimated variance, normal CIs for λ can be computed,^26^^,^^27^^,^^28^ which are expected to work well in terms of the coverage when the estimator $\hat{\lambda }$ is approximately normally distributed.

##### NonPVar: a simple nonparametric estimator of the variance

Instead of imposing a distributional assumption on M, a simple nonparametric method can be considered (NonPVar). NonPVar relies only on the assumption of random partitioning. In particular, NonPVar estimates the variance of $\hat{\lambda }$ as the empirical variance

(Equation 11) $$S_{\lambda }^{2}=\frac{1}{r-1}\sum_{i=1}^{r}{\left({\hat{\lambda }}_{i}-\bar{\lambda }\right)}^{2}\;$$

with $\bar{\lambda }$ the average of the estimates ${\hat{\lambda }}_{i}$. A variance estimator of $\hat{\mu }$ is then given by $S_{\mu }^{2}=\frac{1}{r}S_{\lambda }^{2}$. Note that the sample standard deviation is an underestimation of the standard deviation^32^, so that variance is benchmarked.

CIs can be constructed based on the asymptotic normality of the estimator $\hat{\mu }$, but as an improvement for a small sample size (i.e., small number of replicates r, often r<5), we suggest using quantiles of a t− distribution with r−1 degrees of freedom. In particular, a 1−α CI of μ is obtained as

(Equation 12) $$\left[\hat{\mu }-S_{\mu }t_{r-1;\alpha /2},\hat{\mu }+S_{\mu }t_{r-1;\alpha /2}\right].$$

The significance level α is set at 5% in this paper.

In summary, when there are replicates, BinomVar and NonPVar are generic methods and can be applied to absolute quantification, CNV in singleplex and duplex, fractional abundance and DNA integrity measurement, among other quantities of interest (see Section absolute quantification, Section CNV in singleplex and Section CNV in duplex for the variance estimation of different quantities of interest). NonPVar is expected to be more robust than BinomVar and the delta method when the Poisson assumption is violated (e.g., when additional sources of error or variability are present). However, BinomVar provides more precise variance estimates as it relies on specific distributional assumptions.

#### Proposed methods: Detailed description

##### Probabilistic framework

Suppose the number of molecules in the sample (M) is fixed, as well as the total number of partitions in the dPCR run (n). Then the probability of k partitions being positive is given by

(Equation 13) $$\begin{array}{cc} & P\left(K=k|m,n\right)\\ = & \frac{n!S_{2}\left(m,k\right)}{\left(n-k\right)!n^{m}}\\ = & \frac{n!\left\{\begin{array}{c} m\\ k \end{array}\right\}}{\left(n-k\right)!n^{m}}\\ = & \frac{n!}{\left(n-k\right)!n^{m}}\frac{1}{k!}\sum_{i=0}^{k}{\left(-1\right)}^{i}\left(\begin{array}{c} k\\ i \end{array}\right){\left(k-i\right)}^{m} \end{array}$$

where $S_{2}\left(m,k\right)$ refers to the Stirling number of the second kind, which is the number of ways to partition m objects into k non-empty subsets.^33^ This equation is also mentioned in.^20^ For the sake of computation, $S_{2}\left(m,k\right)$ is approximated as,

(Equation 14) $$\left\{\begin{array}{c} m\\ k \end{array}\right\}∼\sqrt{\frac{v-1}{v\left(1-G\right)}}{\left(\frac{v-1}{v-G}\right)}^{m-k}\frac{k^{m}}{m^{k}}e^{k\left(1-G\right)}\left(\begin{array}{c} m\\ k \end{array}\right)$$

where $G=-W_{0}\left(-ve^{-v}\right),v=m/k$, and $W_{0}\left(z\right)$ is the main branch of the Lambert W function.^34^ Equation 13 is also log-transformed to deal with factorial and power function. The log-transformed probability l of k partitions being positive can be expressed as,

(Equation 15) $$\begin{array}{cc} l= & \sum_{i=1}^{n}\log\left(i\right)-\sum_{i=1}^{n-k}\log\left(i\right)-m\;\log\left(n\right)+\frac{1}{2}\log\left(\frac{v-1}{v\left(1-G\right)}\right)+\left(m-k\right)\log\left(\frac{v-1}{v-G}\right)\\ & +m\;\log\left(k\right)-k\;\log\left(m\right)+k\left(1-G\right)+\sum_{i=1}^{m}\log\left(i\right)-\sum_{i=1}^{m-k}\log\left(i\right)-\sum_{i=1}^{k}\log\left(i\right) \end{array}$$

this equation requires integer n, m and k. Since m and n are considered fixed, this distribution reflects the variability in the number of molecules per partition as a consequence of the random partitioning. Although with this distribution function and with basic probability calculus it is possible to find the variance of $\hat{\lambda }$, these calculations are hard.

Now consider taking a sample from a larger volume (e.g. a specimen). With c the concentration of target molecules in the volume, and with $V_{d}$ the volume to be loaded in the dPCR device, set $\mu =V_{d}c$, i.e. μ is the average number of target molecules loaded in the dPCR device. The random sampling (i.e. pipetting) of a volume $V_{d}$ from the large volume will bring a number of target molecules along; this number of molecules is denoted by $M_{i}$, i=1,…,r, and it is thus considered as a random number.

Taking into account both the random partitioning and sampling, the distribution of $K_{i}$ can be formulated as,

(Equation 16) $$\begin{array}{cc} P\left(K_{i}=k|n_{i}\right) & =\sum_{m=0}^{\infty }P\left(K_{i}=k|M=m,n_{i}\right)P\left(M=m|n_{i}\right)\text{.} \end{array}$$

This would be the appropriate distribution for deriving the standard error of $\hat{\lambda }$, but it can only be used if the distribution of M is known.

Under ideal conditions it would be reasonable to assume a Poisson distribution, i.e.

$$M_{i}∼\text{Poisson}\left(\mu \right)\text{.}$$

See also Figure 1 in the main text. In this setting, the interest is in the estimation of μ (absolute quantification) or a function of μ (e.g. $CNV=\mu_{A}/\mu_{B}$, with $\mu_{A}$ and $\mu_{B}$ the numbers of molecules of type A and B; see further down). Based on a single replicate i, the parameter μ can be estimated as $\frac{V_{d}}{V_{p}}{\hat{\lambda }}_{i}$ with ${\hat{\lambda }}_{i}$ as in Equation 6. With r replicates, the estimator of *μ* can be defined as

(Equation 17) $$\hat{\mu }=\frac{V_{d}}{V_{p}r}\sum_{i=1}^{r}{\hat{\lambda }}_{i}.$$

where $V_{p}$ is the partition volume. The appropriate variance is now $\text{Var}\left\{\hat{\mu }|n_{1},\ldots ,n_{r}\right\}$, which is no longer conditional on the number(s) of molecules, as it must also express the variability over the replicates (replicates involve random sampling from the volume). We will use the shorter notation $\text{Var}\left\{\hat{\mu }|n\right\}$.

For absolute quantification we find

$$\text{Var}\left\{\hat{\mu }|n\right\}={\left(\frac{V_{d}}{V_{p}r}\right)}^{2}\sum_{i=1}^{r}\text{Var}\left\{{\hat{\lambda }}_{i}|n_{i}\right\}\text{.}$$

and thus the problem is reduced to finding the variance $\text{Var}\left\{{\hat{\lambda }}_{i}|n_{i}\right\}$. As before, since $\hat{\lambda }$ is a function of K, we need $\text{Var}\left\{K_{i}|n_{i}\right\}$ and hence the conditional distribution of $K_{i}|n_{i}$.

The conditional distribution of $K_{i}|n_{i}$ can be approximated for large numbers of partitions $n_{i}$. We find from Equation 16,

(Equation 18) $$\begin{array}{cc} P\left(K_{i}=k|n_{i}\right) & =\sum_{m=0}^{\infty }\frac{n_{i}!}{\left(n_{i}-k\right)!n_{i}^{m}}\left\{\begin{array}{l} m\\ k \end{array}\right\}\frac{{\left(n_{i}\lambda_{i}\right)}^{m}}{m!}e^{-n_{i}\lambda_{i}}\\ & =\frac{n_{i}!}{\left(n_{i}-k\right)!}e^{-n_{i}\lambda_{i}}\sum_{m=0}^{\infty }\left\{\begin{array}{l} m\\ k \end{array}\right\}\frac{\lambda_{i}^{m}}{m!}\\ & =\frac{n_{i}!}{\left(n_{i}-k\right)!}e^{-n_{i}\lambda_{i}}\frac{1}{k!}{\left(e^{\lambda_{i}}-1\right)}^{k}\\ & =\frac{n_{i}!}{\left(n_{i}-k\right)!k!}{\left(e^{-\lambda_{i}}\right)}^{n_{i}-k}{\left(1-e^{-\lambda_{i}}\right)}^{k}\text{.} \end{array}$$

We have used the exponential generating function,

$$\lim_{u\to \infty }\sum_{m=0}^{u}\left\{\begin{array}{l} m\\ k \end{array}\right\}\frac{\lambda^{m}}{m!}=\frac{1}{k!}{\left(e^{\lambda }-1\right)}^{k}\text{.}$$

We have the final expression,

(Equation 19) $$P\left(K_{i}=k|n_{i}\right)\overset{\cdot }{∼}\frac{n_{i}!}{\left(n_{i}-k\right)!k!}{\left(e^{-\mu /n_{i}}\right)}^{n_{i}-k}{\left(1-e^{-\mu /n_{i}}\right)}^{k}$$

which is the distribution function of the binomial distribution $\mathrm{Binom}\left(n_{i},\pi_{i}\right)$ with $\pi_{i}=1-e^{-\mu /n_{i}}$ the probability that a partition is positive.

##### Binomial bootstrap process

The parametric bootstrap algorithm works as follows. With B the bootstrap iteration, typically a large number (e.g. B=1000):

1. estimate $\hat{\mu }$ as $\frac{V_{d}}{V_{p}r}\sum_{i=1}^{r}{\hat{\lambda }}_{i}$, with ${\hat{\lambda }}_{i}$ the traditional estimate of $\lambda_{i}$ based on the Poisson approximation. This equation simplifies to the ordinary mean in most cases.
2. set i=1
3. randomly sample B observations from $\mathrm{Binom}\left(n_{i},\pi_{i}\right)$ with $\pi_{i}=1-e^{-\hat{\mu }/n_{i}}$. These are denoted as $k^{b}$, b=1,…,B. For each bootstrap sample b, computer ${\hat{\lambda }}_{i}^{b}$.
4. based on the B estimates ${\hat{\lambda }}_{i}^{b}$, calculate its sample variance ${\hat{\sigma }}_{i}^{2}$
5. if i<r, then i←i+1 and return to step 3; otherwise stop the procedure.
6. average ${\hat{\sigma }}_{i}^{2}$ over the r replicates

With $S_{\lambda }^{2}$ the result of the averaging in step 6, the variance of $\hat{\mu }$ is estimated as $S_{\mu }^{2}=\frac{V_{d}^{2}}{V_{p}^{2}r}S_{\lambda }^{2}$.

##### Delta method for variance estimation

The delta method is a well-established method in statistics for approximating the variance of estimators that are nonlinear functions of the sample observations. It is based on a first order Taylor expansion of this nonlinear function, which essentially “linearises" the function around a point of interest.

This method has been applied to the estimator $\hat{\lambda }$ of Equation 6, resulting in

(Equation 20) $$\text{Var}\left\{\hat{\lambda }|n,\lambda \right\}\approx \frac{\pi }{n\left(1-\pi \right)}$$

with π=1−exp(−λ) the probability of a positive partition. With $\hat{\pi }=1-\exp\left(-\hat{\lambda }\right)=K/n$ an estimator of π, the conditional variance of $\hat{\lambda }$ can be computed by substituting π with $\hat{\pi }$ in Equation 20, resulting in the variance estimator K/[n(n−K)].

Based on the delta method,^28^ gives an approximation of the variance of CNV estimators based on two independent singleplex experiments. The numbers of positive partitions of both the target and reference are considered to follow the binomial distribution (Equation 18). The delta method is applied to the log transformed CNV estimate, resulting in the approximation

(Equation 21) $$\text{Var}\left\{\log\frac{{\hat{\lambda }}_{t}}{{\hat{\lambda }}_{r}}|n,\lambda_{t},\lambda_{r}\right\}\approx \frac{\left(1-\exp\left(-\lambda_{t}\right)\right)}{\left(n\times \lambda_{t}^{2}\;\exp\left(-\lambda_{t}\right)\right)}+\frac{\left(1-\exp\left(-\lambda_{r}\right)\right)}{\left(n\times \lambda_{r}^{2}\;\exp\left(-\lambda_{r}\right)\right)}\text{,}$$

where $\lambda_{t}$ and $\lambda_{r}$ refer to the target and reference, respectively. An estimator of this variance is obtained by replacing the λ parameters by their estimators (Equation 6). A disadvantage of this approach is that it only gives a variance estimate of the log−CNV; it cannot be accurately backtransformed to the original CNV scale. If the estimate is used for the calculation of a CI of the log−CNV, then the boundaries of this interval can be correctly backtransformed to the boundaries of a CI of the CNV by exponentiating these bounds.

The variance $\text{Var}\left\{{\hat{\lambda }}_{i}|n_{i}\right\}$ can now be approximated by using (1) the delta method and (2) the variance $\text{Var}\left\{K_{i}|n_{i}\right\}$ from the binomial distribution (Equation 19). The unknown parameter μ must be replaced by its estimator (Equation 17).

##### General approach for multiplexing

In the next few paragraphs, a more generic description is given, which also applies to multiplex experiments. The description is given for experiments with replicates, but at the end it will be indicated how the procedure simplifies when no replicate is available.

For multiplex experiments, for replicate i=1,…,r, let $M_{Ai},M_{Bi},\ldots$ denote the randomly sampled numbers of target molecules of type A, B, … that are partitioned over the $n_{i}$ partitions. Let $M_{i}^{t}=\left(M_{Ai},M_{Bi},\ldots \right)$, $M^{t}=\left(M_{1}^{t},\ldots ,M_{r}^{t}\right)$ and $n^{t}=\left(n_{1},\ldots ,n_{r}\right)$. The numbers of positive partitions for types of targets A, B, … in replicate i are denoted by $K_{Ai},K_{Bi},\ldots$ Let $K_{i}^{t}=\left(K_{Ai},K_{Bi},\ldots \right)$ and $K^{t}=\left(K_{1}^{t},\ldots ,K_{r}^{t}\right)$. For all types of target molecules, let $\mu_{A},\mu_{B},\ldots$ denote the average numbers of molecules in a fixed volume $V_{d}$ loaded in the dPCR device. The parameters $\lambda_{Ai},\lambda_{Bi},\ldots$ refer to the average numbers of molecules of type A, B, …per partition in replicate i.

Suppose that the goal of the experiment is to estimate a parameter θ which can be expressed as a function of the μ (or λ) parameters. An estimator of θ can be obtained by replacing all μ (or λ) parameters by their estimators, which in turn depend on K and n. It will be convenient to also explicitly consider the estimator $\hat{\theta }$ as a function of M, because the distribution of K depends on it. We therefore write the estimator of θ as $\hat{\theta }=\hat{\theta }\left(M,K,n\right)$. We are now interested in the estimation of its variance, ${\text{Var}}_{MX}\left\{\hat{\theta }|n\right\}$, which is estimated as the empirical variance over the replicates and covers both sampling and random partitioning variability.

In the special case of no replicates (i.e. r=1), we cannot make a distinction between the concentration of target molecules in the vessel and the concentration of the target loaded on the dPCR device. Hence, λ takes over the role of μ, and the number of target molecules is considered fixed, i.e. we use m instead of M. This number is no longer considered random and so we only need the conditional variance ${\text{Var}}_{K|M}\left\{\hat{\theta }|M=m,n\right\}$. The estimation of this conditional variance is based on K which has the distribution function in Equation 13.

##### Absolute quantification

For absolute quantification, the target parameter is the average number of copies per partition λ. Here, only one type of target molecules needs to be quantified (see Figure 2A). We also allow for technical replicates.

For all replicates i=1,…,r, we can calculate ${\hat{\lambda }}_{i}$ from $K_{i}$ as in Equation 6 in SI. Since we have replicates, the final estimator of λ becomes $\hat{\lambda }=\frac{1}{r}\sum_{i=1}^{r}{\hat{\lambda }}_{i}$ with variance

(Equation22) $${\text{Var}}_{KM}\left\{\hat{\lambda }\right\}=\frac{1}{r^{2}}\sum_{i=1}^{r}{\text{Var}}_{KM}\left\{{\hat{\lambda }}_{i}|n_{i}\right\}.$$

For the estimation of ${\text{Var}}_{KM}\left\{{\hat{\lambda }}_{i}\right\}$ we use the BinomVar procedure as in Section BinomVar: binomial bootstrap for variances or non-parametrically estimate the between-replicate variability of $\hat{\lambda }$ with the NonPVar method as described in Section NonPVar: a simple nonparametric estimator of the variance.

##### CNV in singleplex

BinomVar and NonPVar can also be applied to CNV estimation. CNV is defined as large-scale losses and gains of DNA fragments and is one of the major classes of genetic variation.^35^ It quantifies how the number of copies of a target gene varies from a reference. In a CNV singleplex set-up, the target (A) and reference (B) molecules are quantified in separate experiments (Figure 2B).

We now consider the estimators

(Equation 23) $${\hat{CNV}}_{ij}=\hat{CNV}\left(K_{Ai},M_{Ai},K_{Bj},M_{Bj}\right)=\frac{{\hat{\mu }}_{Ai}}{{\hat{\mu }}_{Bj}}=\frac{{\hat{\lambda }}_{Ai}}{{\hat{\lambda }}_{Bj}}\frac{V_{di}/V_{pi}}{V_{dj}/V_{pj}}$$

based on replicate i (j) for molecule A (B). Very often it is reasonable to have $V_{di}$ equal to $V_{dj}$ and $V_{pi}$ equal to $V_{pj}$. It will turn out to be convenient if we estimate the log−CNV instead,

(Equation 24) $${\hat{\theta }}_{ij}=\hat{\theta }\left(X_{Ai},M_{Ai},X_{Bj},M_{Bj}\right)=\ln\frac{{\hat{\lambda }}_{Ai}}{{\hat{\lambda }}_{Bj}}=\ln\;{\hat{\lambda }}_{Ai}-\ln\;{\hat{\lambda }}_{Bj}.$$

The final estimator of the log−CNV is given by (assuming equal numbers of replicates)

(Equation 25) $$\hat{\theta }=\frac{1}{r^{2}}\sum_{i=1}^{r}\sum_{j=1}^{r}{\hat{\theta }}_{ij}=\frac{1}{r}\sum_{i=1}^{r}\left(\ln\;{\hat{\lambda }}_{Ai}-\ln\;{\hat{\lambda }}_{Bi}\right).$$

Upon relying on the independence between the singleplex experiments, the variance of $\hat{\theta }$ is given by

(Equation 26) $${\text{Var}}_{KM}\left\{\hat{\theta }\right\}=\frac{1}{r^{2}}\sum_{i=1}^{r}{\text{Var}}_{KM}\left\{\ln\;{\hat{\lambda }}_{Ai}|n_{i}\right\}+\frac{1}{r^{2}}\sum_{i=1}^{r}{\text{Var}}_{KM}\left\{\ln\;{\hat{\lambda }}_{Bi}|n_{i}\right\}\text{.}$$

For both terms we apply the same procedures as in Section absolute quantification.

##### CNV in duplex

In the CNV duplex set-up, the target and reference are typically quantified in the same dPCR run and thus the number of partitions is the same and other sources of errors are shared. There is matching between the replicates for A and B (see Figure 2B). We consider the estimator

(Equation 27) $${\hat{CNV}}_{i}=\hat{CNV}\left(K_{Ai},M_{Ai},K_{Bi},M_{Bi}\right)=\frac{{\hat{\lambda }}_{Ai}}{{\hat{\lambda }}_{Bi}}$$

based on replicate i for molecules A and B. With the r replicates, the final estimator becomes $\hat{\text{CNV}}=\frac{1}{r}\sum_{i=1}^{r}{\hat{\text{CNV}}}_{i}$ with variance

(Equation 28) $${\text{Var}}_{KM}\left\{\hat{CNV}\right\}=\frac{1}{r^{2}}\sum_{i=1}^{r}{\text{Var}}_{KM}\left\{{\hat{CNV}}_{i}|n_{i}\right\}.$$

the same procedures as in Section absolute quantification can also be applied here.

#### Simulation study and empirical data analysis

BinomVar and NonPVar were evaluated and compared to competitor methods in a simulation study and a case study. Simulations of the number of positive partitions under a variety of circumstances allow method benchmarking. The case study includes empirical data with replicates of CNV where all methods are compared, as well as fractional abundance data where only BinomVar and NonPVar can be applied.

In the simulation study, we used the simulation pipeline of.^17^ In a first scenario, the number of molecules M is randomly sampled from a Poisson distribution, and, next, given M, the number of positive partitions is generated by random partitioning of the molecules over n partitions. Subsequent scenarios add additional sources of variation and bias to the data generating process as in.^17^ More specifically, we simulate the process for several orders of magnitude of concentration reflecting empirical dilution levels. Therefore we vary the expected number of target molecules per partition λ from 0.005 to 1.5. The pipetting error we added is normally distributed with a coefficient of variation of 3%. The number of partitions is initially set at 20 000. Partitions are assumed to be lost completely at random. To simulate this process, we randomly retain partitions between replicates with an expected value 16000 and standard deviation 2000. Then the partition size is modeled to follow a log− normal distribution with mean 0 and standard deviation 0.1, which is approximately equal to a normal distribution with a coefficient of variation of 10%. In the final stage, partitions are classified as positive or negative after thresholding. To assess the effect of misclassification, we set 5% false negative rate and 0.01% false positive rate. Those simulation studies are implemented in parallel (separately) and sequentially.

For each scenario, the methods were evaluated using 1000 simulation runs, and within each run, 3 replicates were generated. The bootstrap method (in BinomVar) was applied with 1000 bootstrap samples for each replicate. The performance of the variance estimators is evaluated in terms of the bias (relative bias w.r.t. the true variance $\frac{E\left(\hat{\theta }\right)-\theta }{\theta }$ or absolute bias $E\left(\hat{\theta }\right)-\theta$), and the empirical coverage of the 95% CIs (i.e., the relative frequency, over the 1000 simulation runs, that the true quantities of interest falls within the CI) is assessed.

Method performance was evaluated for absolute quantification and CNV, both in singleplex and duplex, and fractional abundance of a mutation and DNA integrity, all in duplex. As competitor methods we included the delta method and the GLMM^26^ methods for absolute quantification and CNV in singleplex and duplex. To our knowledge no competing methods exist for fractional abundance and DNA integrity estimation, unless one would use the delta method for developing expressions for these quantities, which is analytically difficult.

In the case study, we investigated two types of empirical data: CNV in singleplex and mutations in duplex. The mutation data comes from.^8^ Three types of samples were included: (i) patient samples with a very high mutation load (samples 1–13); (ii) homoplasmic wild-type samples from a healthy volunteer (samples 14–16); and (iii) samples undergoing nuclear transfer, thus carrying a low mutation load due to mtDNA carry-over (samples 17–23). The CNV dataset is from^26^ which consists of 10 samples with chromosomal abnormalities and 4 controls. For each sample and gene of interest, there are 2 or 3 technical replicates.

### Quantification and statistical analysis

The data analysis was conducted using R (version 4.2.0).^36^ For fractional abundance data, 6 samples, each with three technical replicates, were analyzed. The detailed results and CIs are presented in Figure 4. For CNV data, 2 samples, each with 2-3 replicates, were analyzed. The corresponding data details and CIs are shown in Figures 3 and S63.
