# Supplementary material for: Flexible methods for uncertainty estimation of digital PCR data
Source: iScience. 2025 Jan 8;28(3):111772. doi: 10.1016/j.isci.2025.111772 (PMC11914197; doi:10.1016/j.isci.2025.111772)
Supplement: Document S1. Figures S1–S63 and Table S1 [file mmc1.pdf]

iScience, Volume *28*

Supplemental information

Flexible methods for uncertainty estimation of digital PCR data

Yao Chen, Ward De Spiegelaere, Matthijs Vynck, Wim Trypsteen, David Gleerup, Jo Vandesompele, and Olivier Thas

Flexible Methods for Uncertainty Estimation of Digital PCR Data

1 dPCR designs

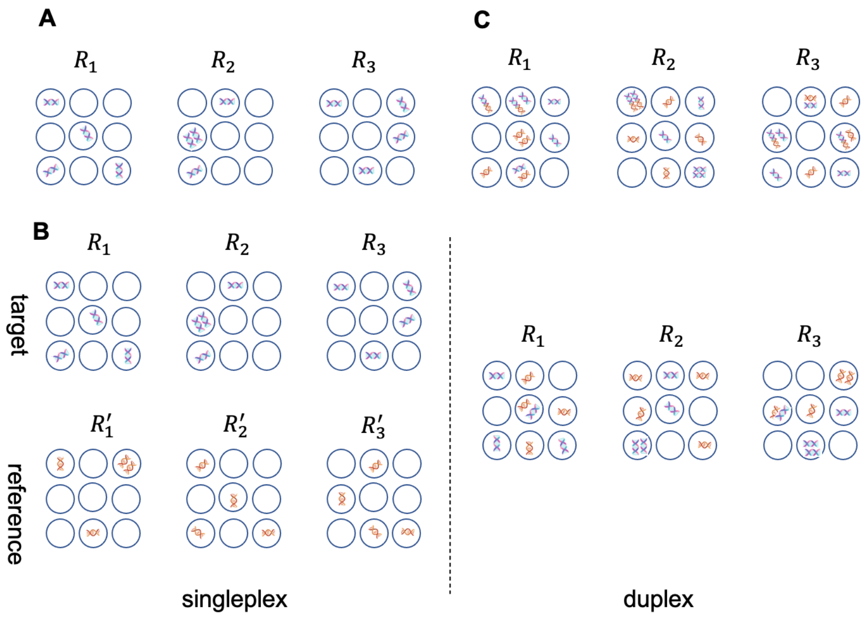

Figure S1: Illustration of the dPCR designs considered in the simulation study, related to STAR Methods. (A) absolute quantification, (B) CNV with singleplex and duplex, and fractional abundance of a mutation in duplex setting, (C) DNA integrity. R_1 up to R_3 refer to replicated reactions.

2 Absolute Quantification

2.1 Relative Bias

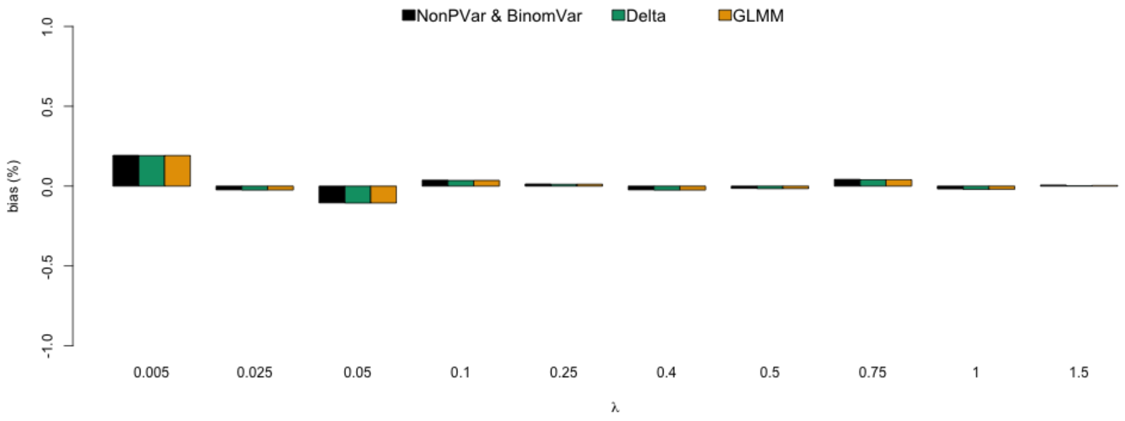

Figure S2: Relative bias of the estimators of λ in absolute quantification (3 replicates) with only sampling variation and random partitioning, related to Fig. 1.

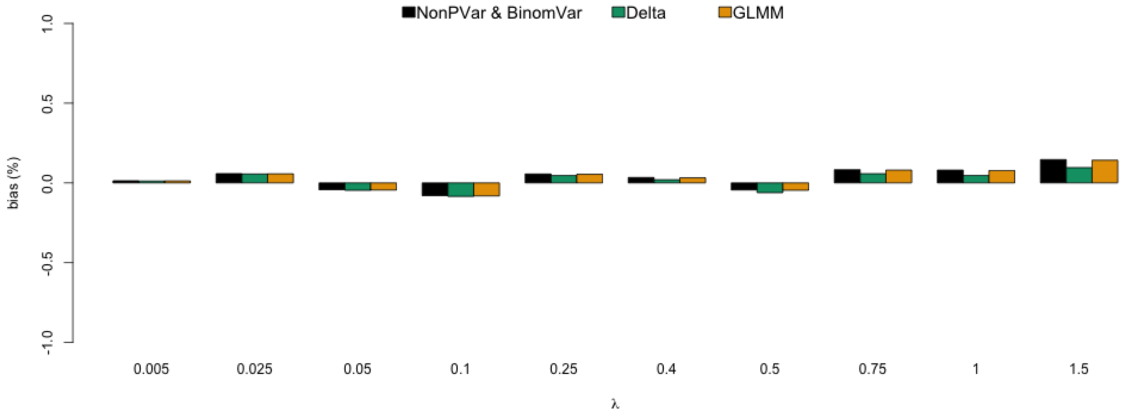

Figure S3: Relative bias of the estimator of λ in absolute quantification (3 replicates) with additional pipetting error, related to Fig. 1.

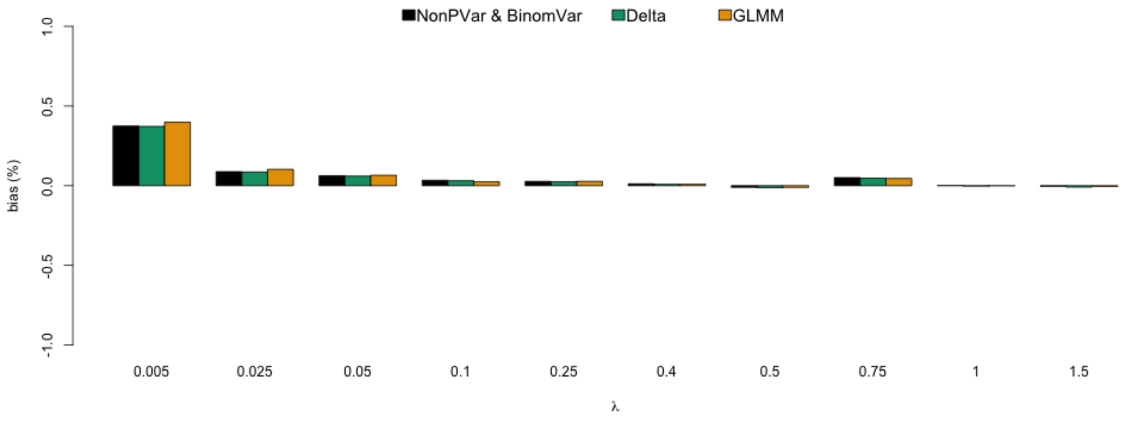

Figure S4: Relative bias of the estimator of λ in absolute quantification (3 replicates) with additional partition loss, related to Fig. 1.

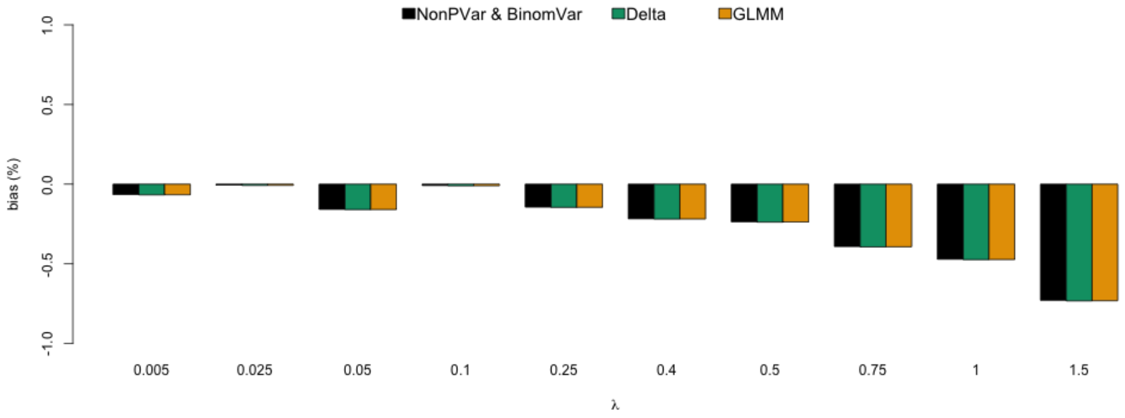

Figure S5: Relative bias of the estimator of λ in absolute quantification (3 replicates) with additional partition size variation, related to Fig. 1.

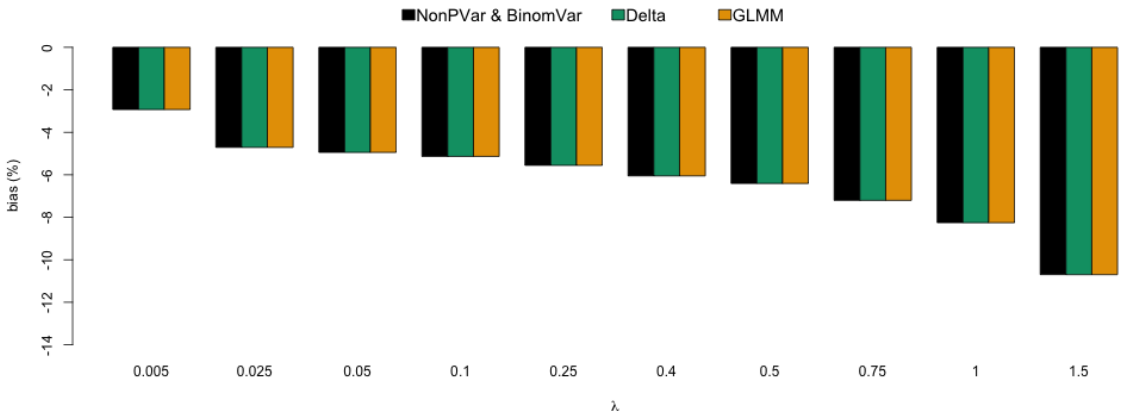

Figure S6: Relative bias of the estimator of λ in absolute quantification (3 replicates) with additional misclassification, related to Fig. 1.

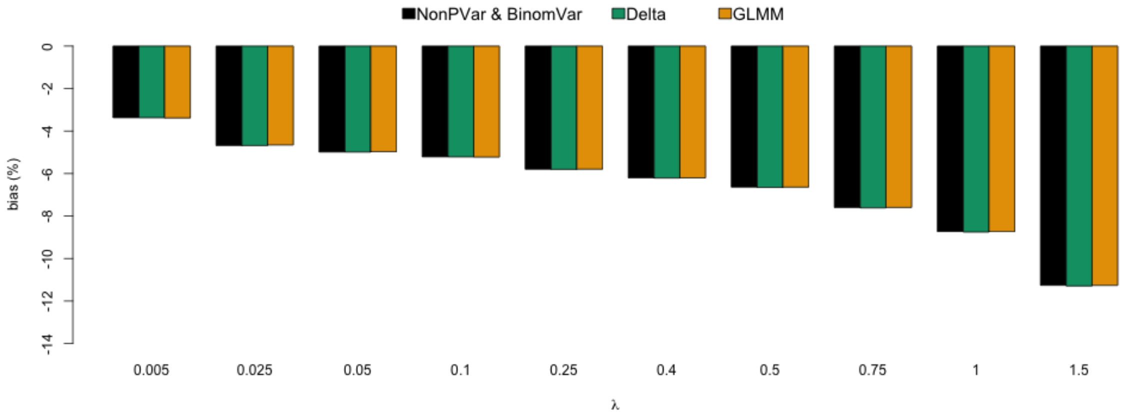

Figure S7: Relative bias of the estimator of λ in absolute quantification (3 replicates) with all sources of variation, related to Fig. 1.

The absolute bias is calculated as,

$$\text{bias(log)} = \log\left(|Var(\hat{\lambda}) - Var(\lambda)|\right)$$

2.2 Absolute Bias

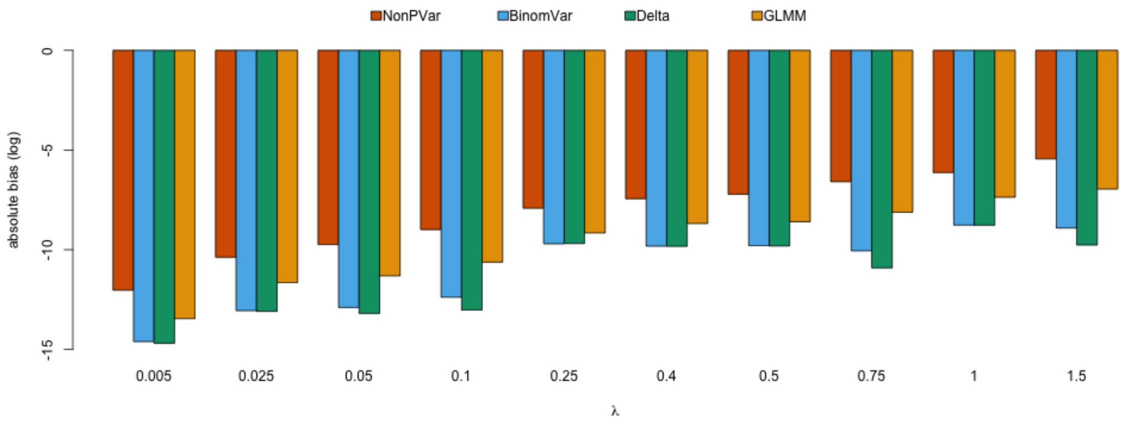

Figure S8: Absolute bias of the variance estimator of λ in absolute quantification (3 replicates) with only sampling variation and random partitioning, related to Fig. 1. Note the absolute bias is on the log scale. Smaller on the log scale means closer to 0 on the original scale. Opposite to the relative bias plots, methods with longer bars (extending further down) are better.

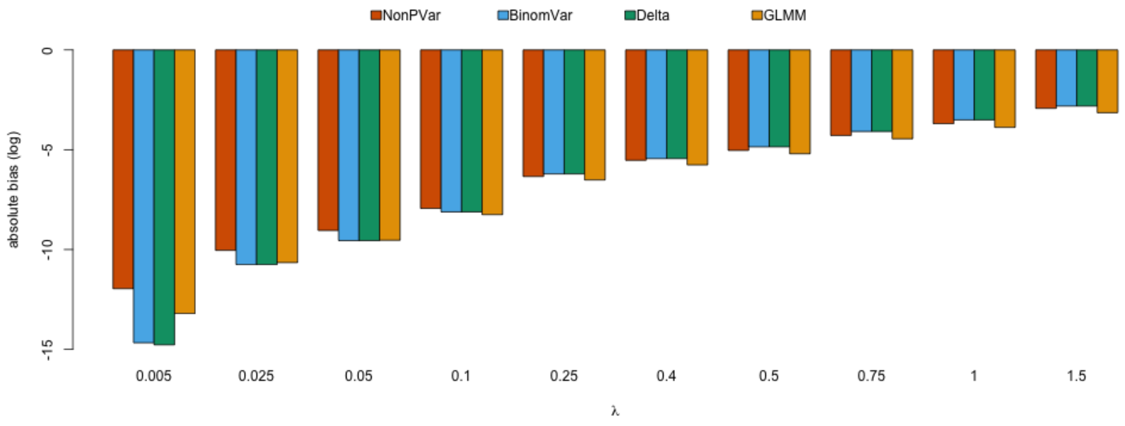

Figure S9: Absolute bias of the variance estimator of λ in absolute quantification (3 replicates) with additional pipetting error, related to Fig. 1. Note the absolute bias is on the log scale. Smaller on the log scale means closer to 0 on the original scale. Opposite to the relative bias plots, methods with longer bars (extending further down) are better.

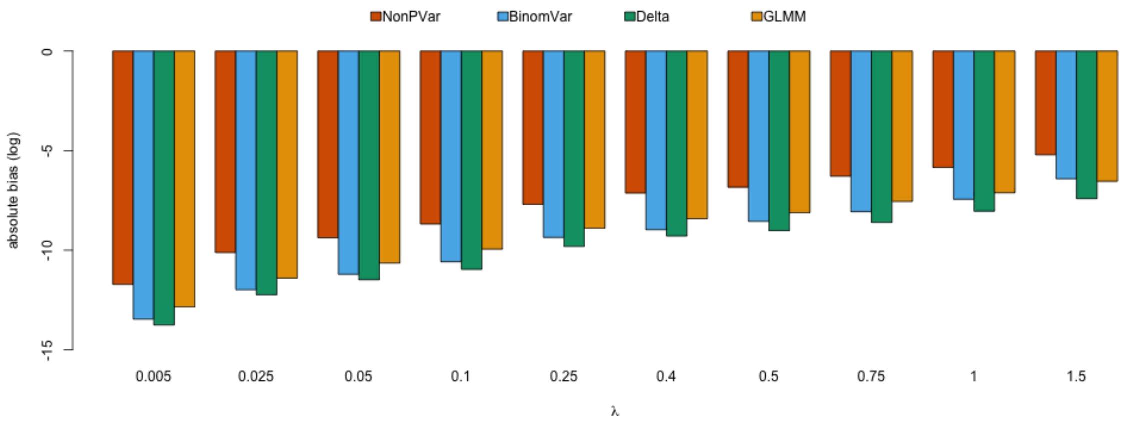

Figure S10: Absolute bias of the variance estimator of λ in absolute quantification (3 replicates) with additional partition loss, related to Fig. 1. Note the absolute bias is on the log scale. Smaller on the log scale means closer to 0 on the original scale. Opposite to the relative bias plots, methods with longer bars (extending further down) are better.

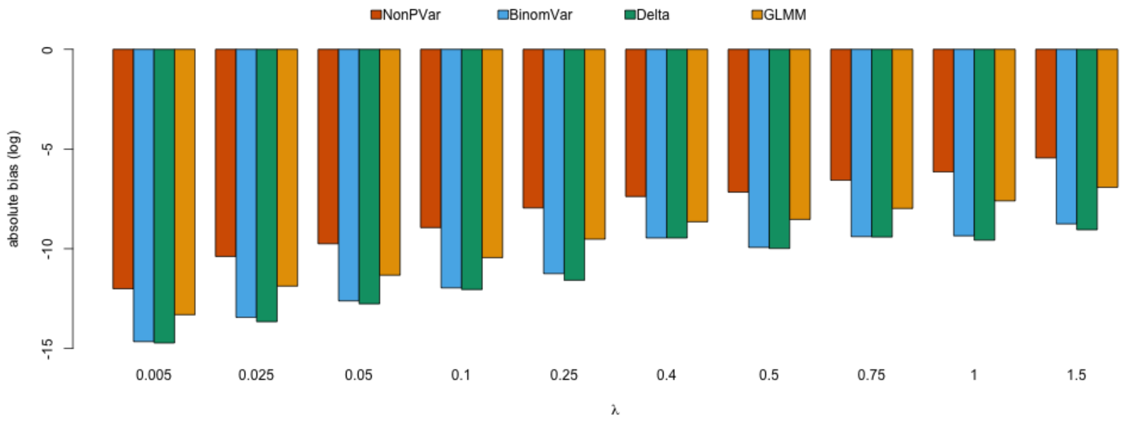

Figure S11: Absolute bias of the variance estimator of λ in absolute quantification (3 replicates) with additional partition size variation, related to Fig. 1. Note the absolute bias is on the log scale. Smaller on the log scale means closer to 0 on the original scale. Opposite to the relative bias plots, methods with longer bars (extending further down) are better.

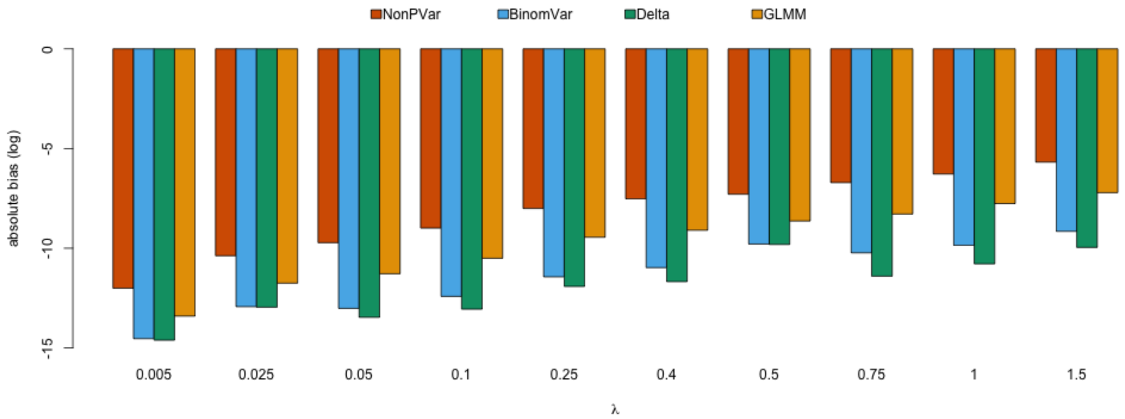

Figure S12: Absolute bias of the variance estimator of λ in absolute quantification (3 replicates) with additional misclassification, related to Fig. 1. Note the absolute bias is on the log scale. Smaller on the log scale means closer to 0 on the original scale. Opposite to the relative bias plots, methods with longer bars (extending further down) are better.

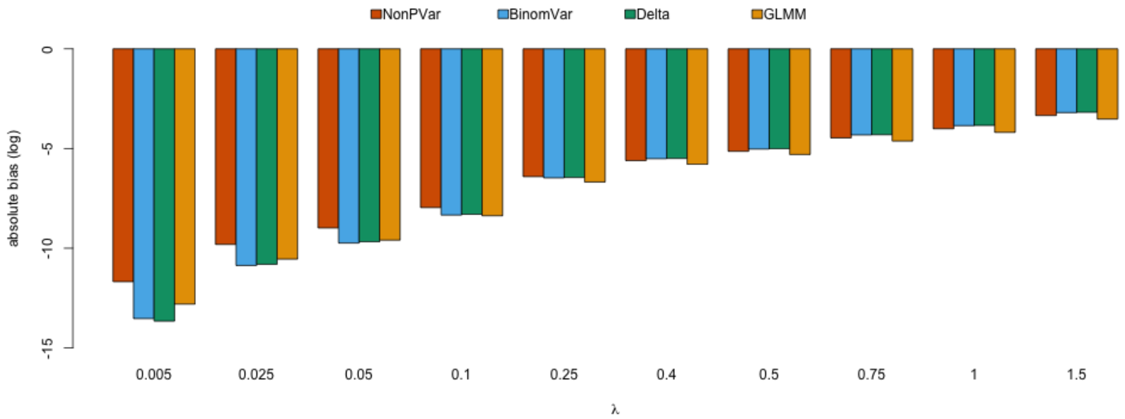

Figure S13: Absolute bias of the variance estimator of λ in absolute quantification (3 replicates) with all sources of variation, related to Fig. 1. Note the absolute bias is on the log scale. Smaller on the log scale means closer to 0 on the original scale. Opposite to the relative bias plots, methods with longer bars (extending further down) are better.

2.3 Variance Estimates

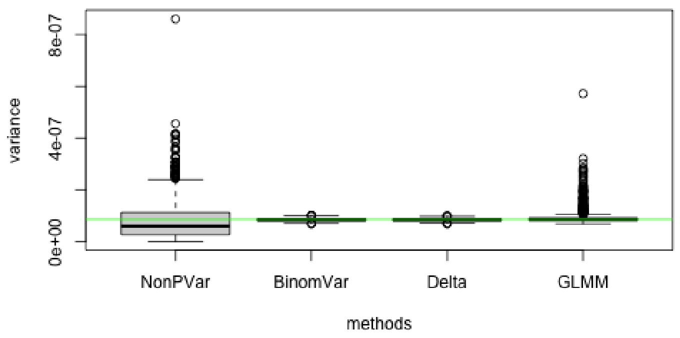

Figure S14: Variance estimates of simulation runs in absolute quantification (3 replicates) with only sampling variation and random partitioning in low concentration setting of $\lambda_A = 0.005$, related to Fig. 1. The horizontal line is the estimated variance in the simulation (a good approximation to the true variance).

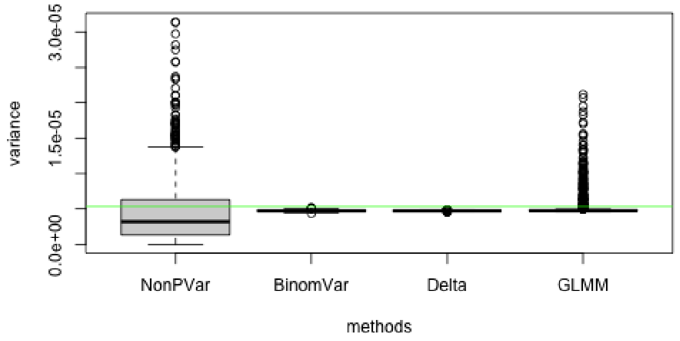

Figure S15: Variance estimates of simulation runs in absolute quantification (3 replicates) with only sampling variation and random partitioning in medium concentration setting of $\lambda_A = 0.25$, related to Fig. 1. The horizontal line is the estimated variance in the simulation (a good approximation to the true variance).

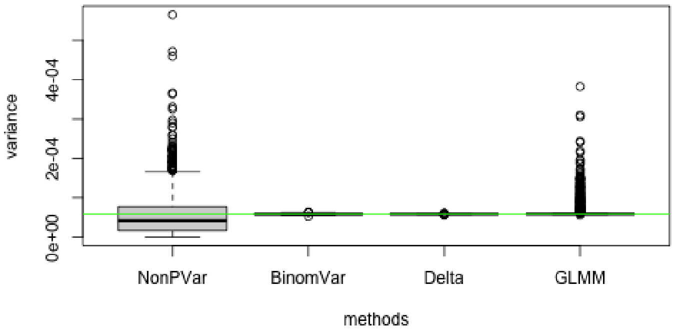

Figure S16: Variance estimates of simulation runs in absolute quantification (3 replicates) with only sampling variation and random partitioning in high concentration setting of $\lambda_A = 1.5$, related to Fig. 1. The horizontal line is the estimated variance in the simulation (a good approximation to the true variance).

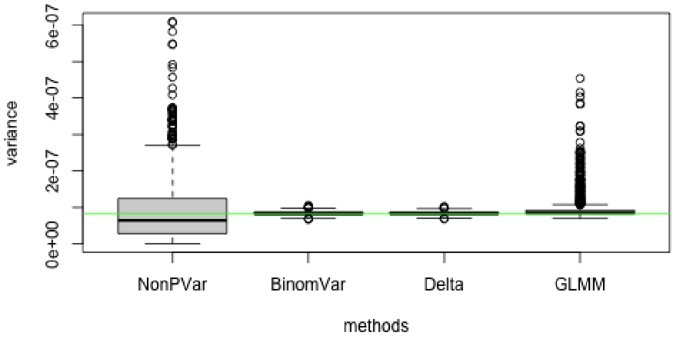

Figure S17: Variance estimates of simulation runs in absolute quantification (3 replicates) with additional pipetting error in low concentration setting of $\lambda_A = 0.005$, related to Fig. 1. The horizontal line is the estimated variance in the simulation (a good approximation to the true variance).

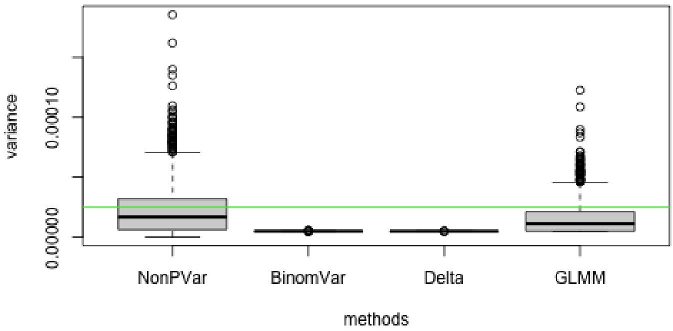

Figure S18: Variance estimates of simulation runs in absolute quantification (3 replicates) with additional pipetting error in medium concentration setting of $\lambda_A = 0.25$, related to Fig. 1. The horizontal line is the estimated variance in the simulation (a good approximation to the true variance).

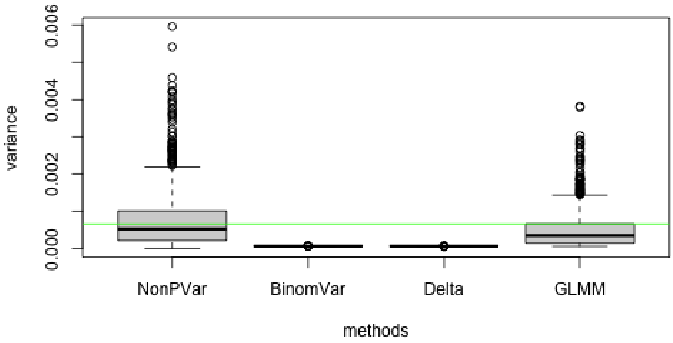

Figure S19: Variance estimates of simulation runs in absolute quantification (3 replicates) with additional pipetting error in high concentration setting of $\lambda_A = 1.5$, related to Fig. 1. The horizontal line is the estimated variance in the simulation (a good approximation to the true variance).

3 Copy Number Variation

3.1 Singleplex

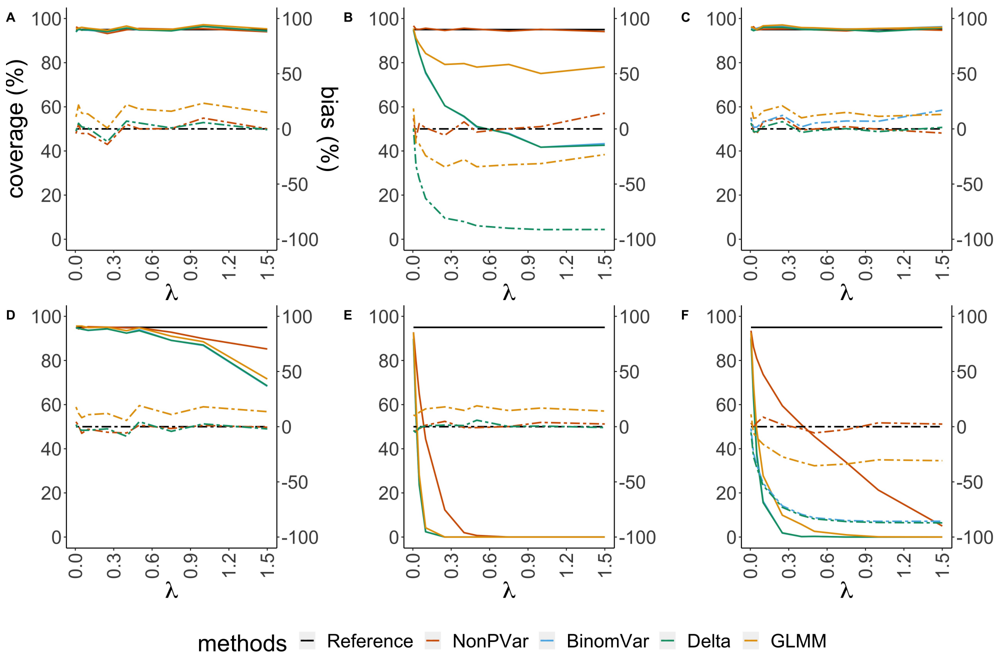

Figure S20: Empirical coverage of the 95% CIs (solid lines, left axis) and relative bias (dashed lines, right axis) for CNV in singleplex in different scenarios, related to Fig. 4. X-axis represents varying concentration of target molecules from low to high. (A) only sampling variation and random partitioning with 3 replicates (B) 3% pipetting error (C) 20% partition loss (D) coefficient of variation of 10% in partition size (E) misclassification with 0.01% false positive rate and 5% false negative rate (F) all variation included. The reference (black solid line) for empirical coverage is set at 95%. The constructed CIs are supposed to cover the true values in 95% of the time. The closer other solid lines are to this reference, the better the CIs are. The reference (black dashed line) is set at 0%. The closer other dashed lines are to this reference, the lower the relative bias is.

3.1.1 Relative Bias

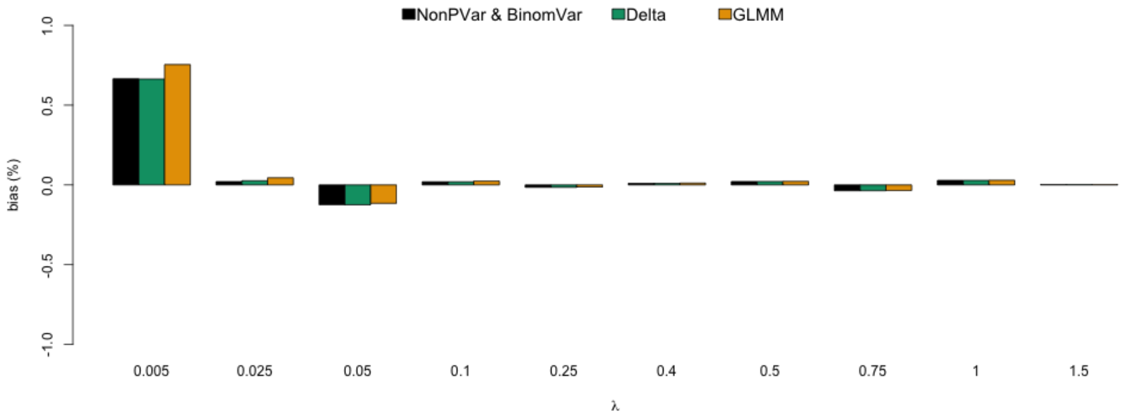

Figure S21: Relative bias of the estimator of cnv in CNV singleplex (3 replicates) with only sampling variation and random partitioning, related to Fig. 4.

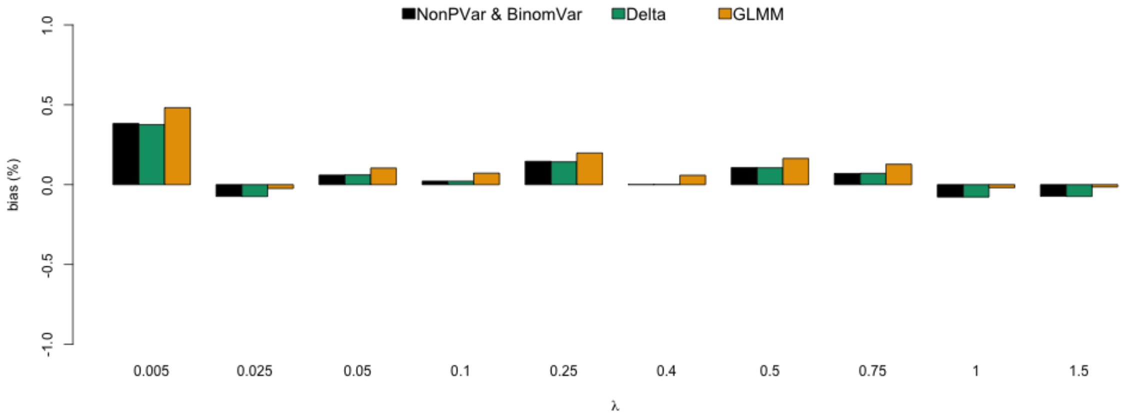

Figure S22: Relative bias of the estimator of cnv in CNV singleplex (3 replicates) with additional pipetting error, related to Fig. 4.

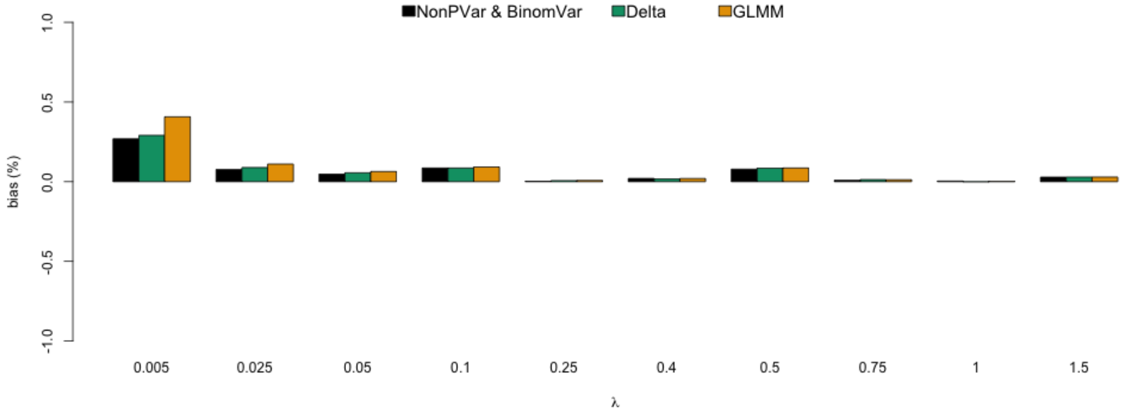

Figure S23: Relative bias of the estimator of cnv in CNV singleplex (3 replicates) with additional partition loss, related to Fig. 4.

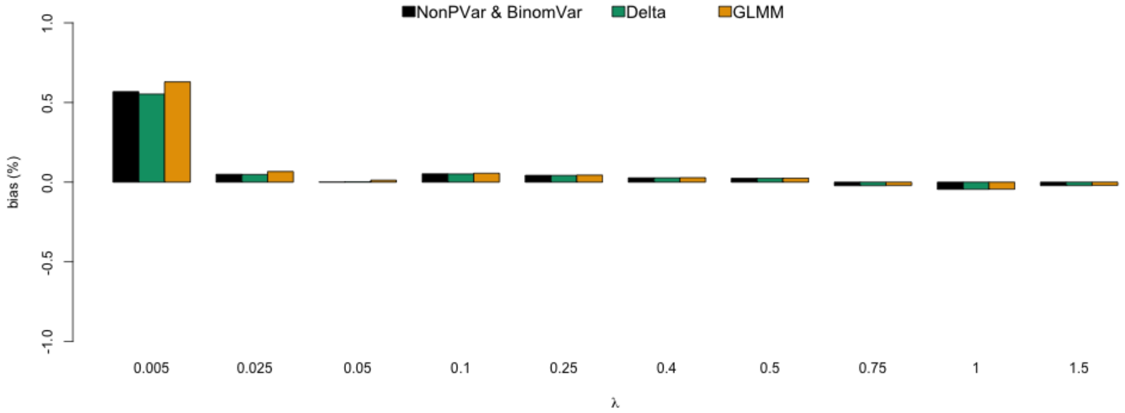

Figure S24: Relative bias of the estimator of cnv in CNV singleplex (3 replicates) with additional partition size variation, related to Fig. 4.

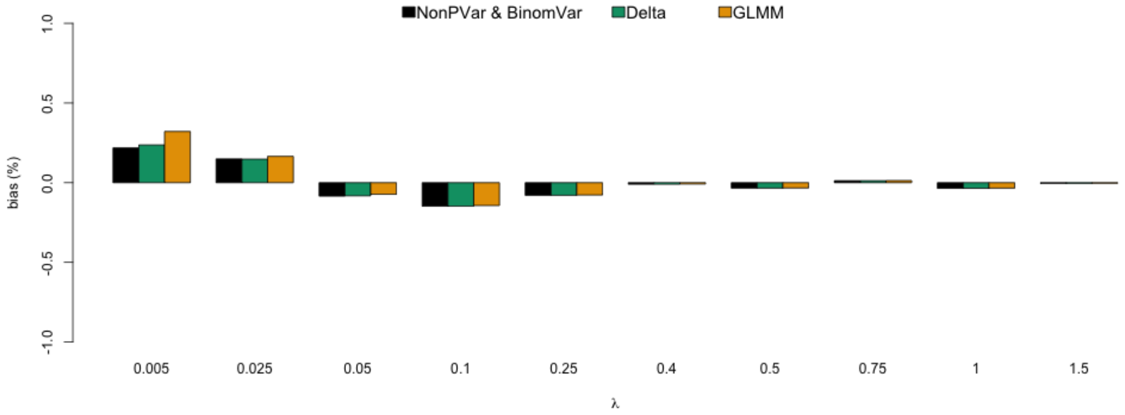

Figure S25: Relative bias of the estimator of cnv in CNV singleplex (3 replicates) with additional misclassification, related to Fig. 4.

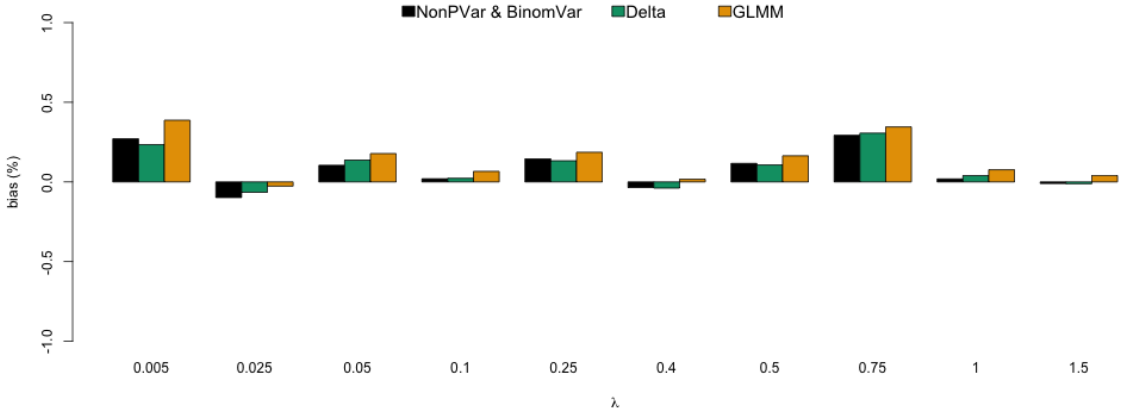

Figure S26: Relative bias of the estimator of cnv in CNV singleplex (3 replicates) with all sources of variation, related to Fig. 4.

3.1.2 Absolute Bias

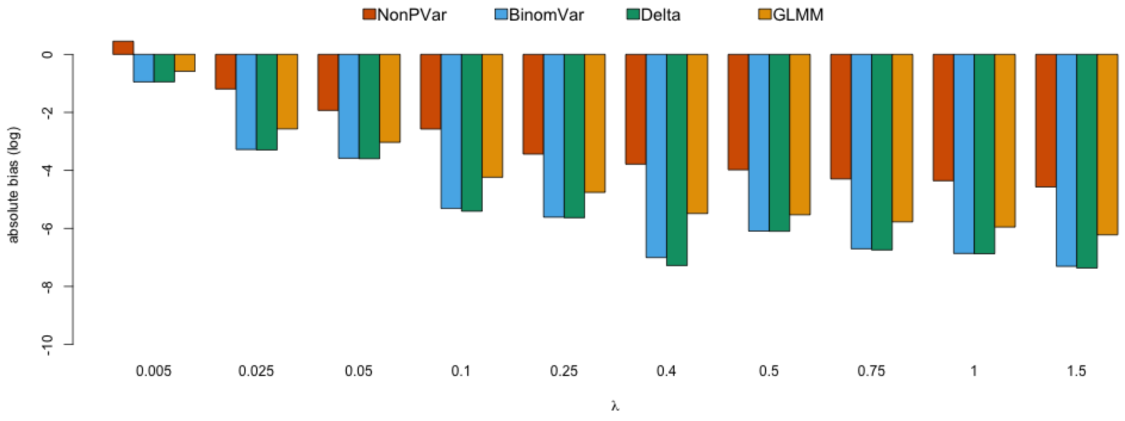

Figure S27: Absolute bias of the variance estimator of cnv in CNV singleplex (3 replicates) with only sampling variation and random partitioning, related to Fig. 4. Note the absolute bias is on the log scale. Smaller on the log scale means closer to 0 on the original scale. Opposite to the relative bias plots, methods with longer bars (extending further down) are better.

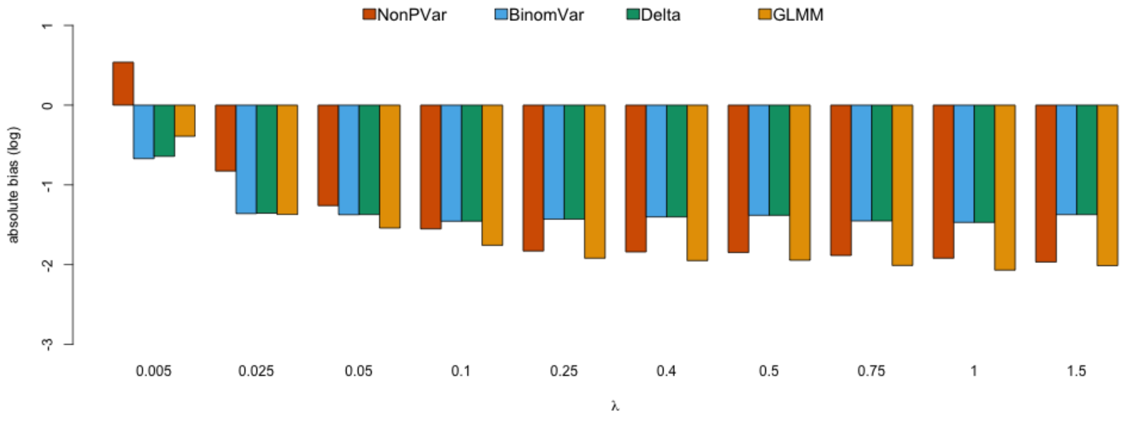

Figure S28: Absolute bias of the variance estimator of cnv in CNV singleplex (3 replicates) with additional pipetting error, related to Fig. 4. Note the absolute bias is on the log scale. Smaller on the log scale means closer to 0 on the original scale. Opposite to the relative bias plots, methods with longer bars (extending further down) are better.

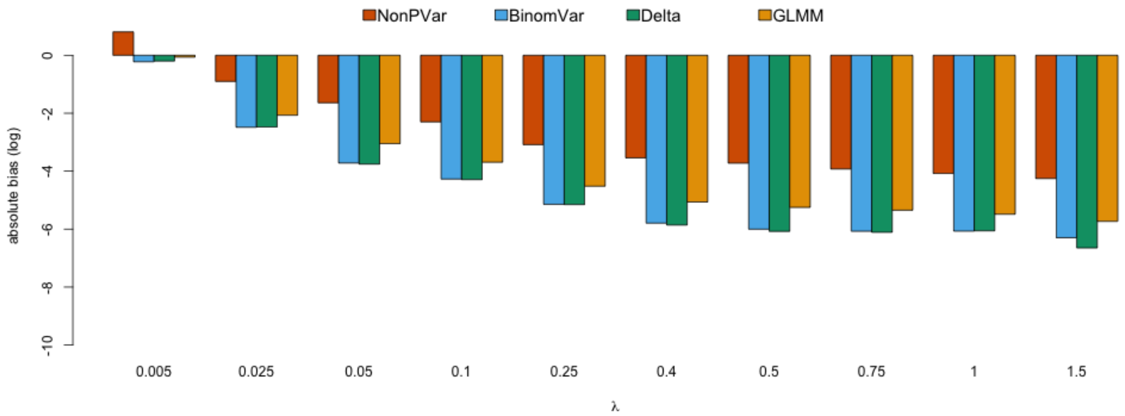

Figure S29: Absolute bias of the variance estimator of cnv in CNV singleplex (3 replicates) with additional partition loss, related to Fig. 4. Note the absolute bias is on the log scale. Smaller on the log scale means closer to 0 on the original scale. Opposite to the relative bias plots, methods with longer bars (extending further down) are better.

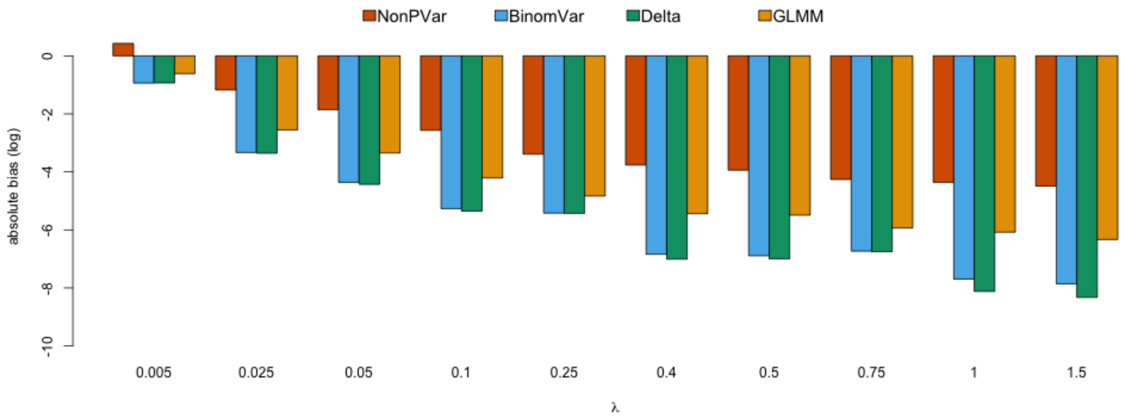

Figure S30: Absolute bias of the variance estimator of cnv in CNV singleplex (3 replicates) with additional partition size variation, related to Fig. 4. Note the absolute bias is on the log scale. Smaller on the log scale means closer to 0 on the original scale. Opposite to the relative bias plots, methods with longer bars (extending further down) are better.

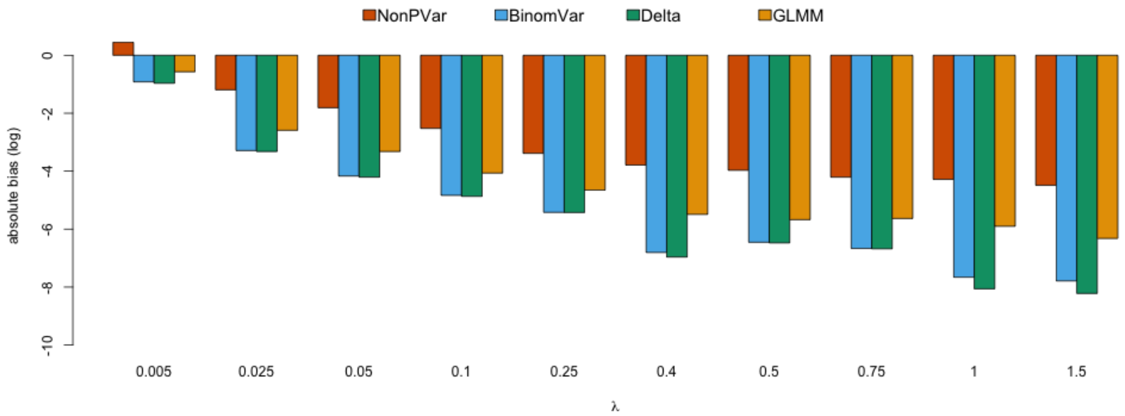

Figure S31: Absolute bias of the variance estimator of cnv in CNV singleplex (3 replicates) with additional misclassification, related to Fig. 4. Note the absolute bias is on the log scale. Smaller on the log scale means closer to 0 on the original scale. Opposite to the relative bias plots, methods with longer bars (extending further down) are better.

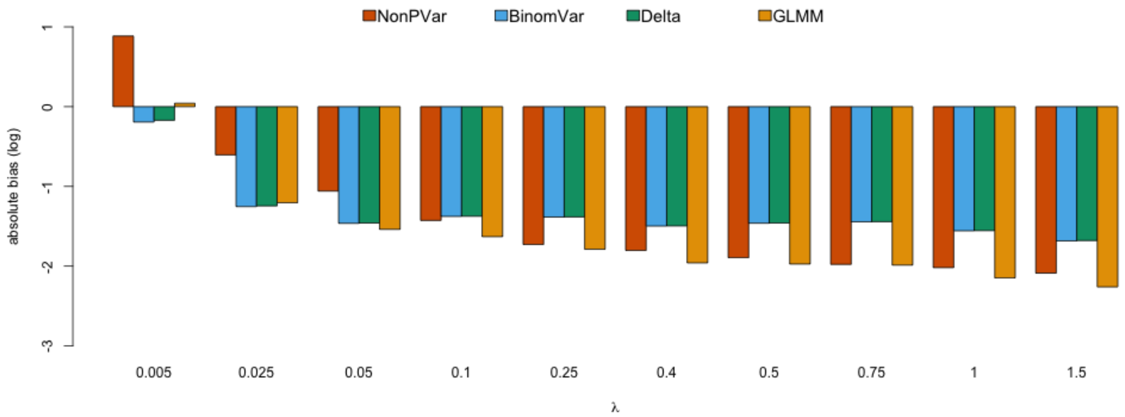

Figure S32: Absolute bias of the variance estimator of cnv in CNV singleplex (3 replicates) with all sources of variation, related to Fig. 4. Note the absolute bias is on the log scale. Smaller on the log scale means closer to 0 on the original scale.

3.1.3 Variance Estimates

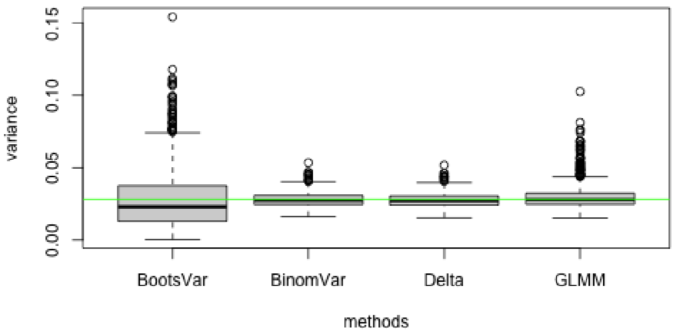

Figure S33: Variance estimates of simulation runs in CNV singleplex (3 replicates) with only sampling variation and random partitioning in low concentration setting of $\lambda_A = 0.005$, related to Fig. 4. The horizontal line is the estimated variance in the simulation (a good approximation to the true variance).

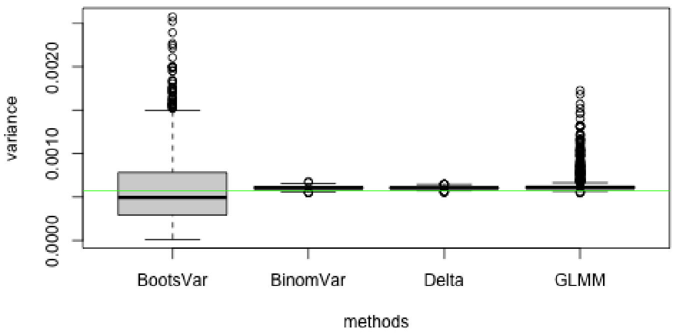

Figure S34: Variance estimates of simulation runs in CNV singleplex (3 replicates) with only sampling variation and random partitioning in medium concentration setting of $\lambda_A = 0.25$, related to Fig. 4. The horizontal line is the estimated variance in the simulation (a good approximation to the true variance).

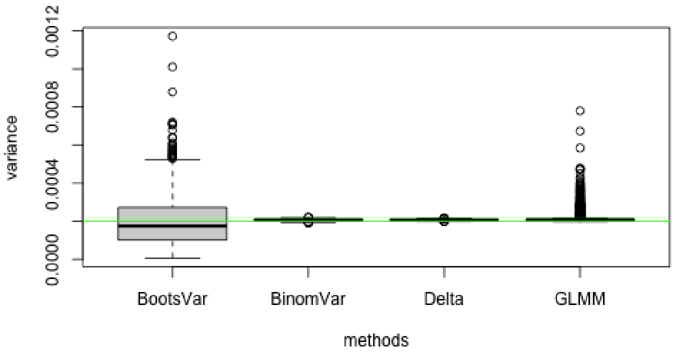

Figure S35: Variance estimates of simulation runs in CNV singleplex (3 replicates) with only sampling variation and random partitioning in high concentration setting of $\lambda_A = 1.5$, related to Fig. 4. The horizontal line is the estimated variance in the simulation (a good approximation to the true variance).

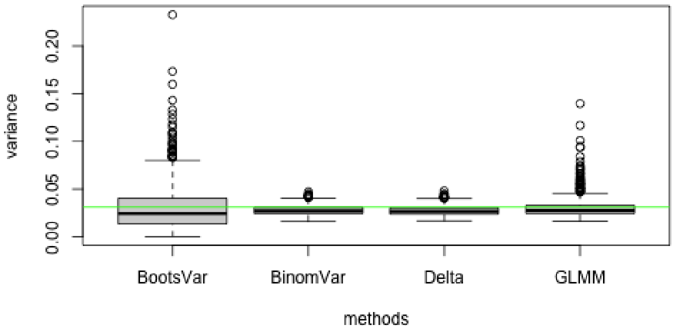

Figure S36: Variance estimates of simulation runs in CNV singleplex (3 replicates) with additional pipetting error in low concentration setting of $\lambda_A = 0.005$, related to Fig. 4. The horizontal line is the estimated variance in the simulation (a good approximation to the true variance).

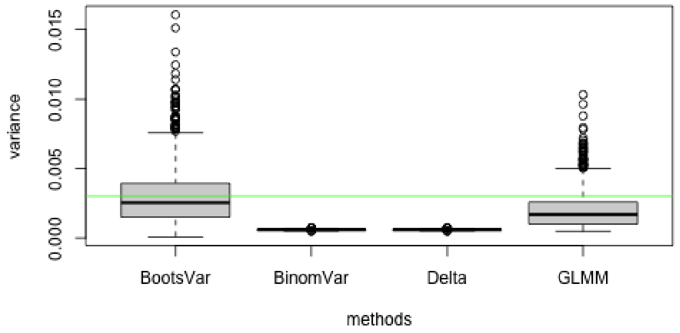

Figure S37: Variance estimates of simulation runs in CNV singleplex (3 replicates) with additional pipetting error in medium concentration setting of $\lambda_A = 0.25$, related to Fig. 4. The horizontal line is the estimated variance in the simulation (a good approximation to the true variance).

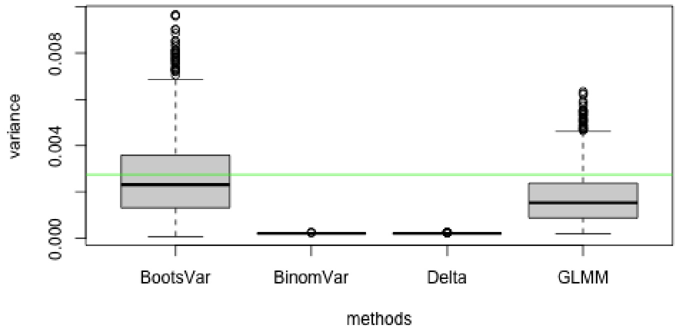

Figure S38: Variance estimates of simulation runs in CNV singleplex (3 replicates) with additional pipetting error in high concentration setting of $\lambda_A = 1.5$, related to Fig. 4. The horizontal line is the estimated variance in the simulation (a good approximation to the true variance).

3.2 Duplex

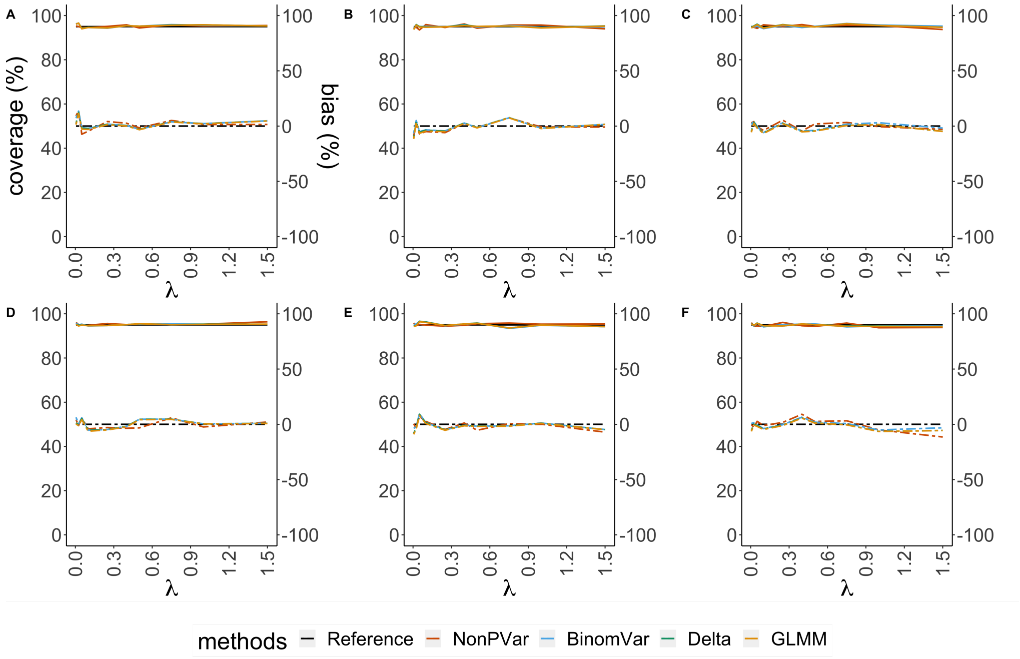

Figure S39: Empirical coverage of the 95% CIs (solid lines, left axis) and relative bias (dashed lines, right axis) for CNV in duplex in different scenarios, related to Fig. 4. X-axis represents varying concentration of target molecules from low to high. (A) only sampling variation and random partitioning with 3 replicates (B) 3% pipetting error (C) 20% partition loss (D) coefficient of variation of 10% in partition size (E) misclassification with 0.01% false positive rate and 5% false negative rate (F) all variation included. The reference (black solid line) for empirical coverage is set at 95%. The constructed CIs are supposed to cover the true values in 95% of the time. The closer other solid lines are to this reference, the better the CIs are. The reference (black dashed line) is set at 0%. The closer other dashed lines are to this reference, the lower the relative bias is.

4 Mutational Load

4.1 Low Mutational Load

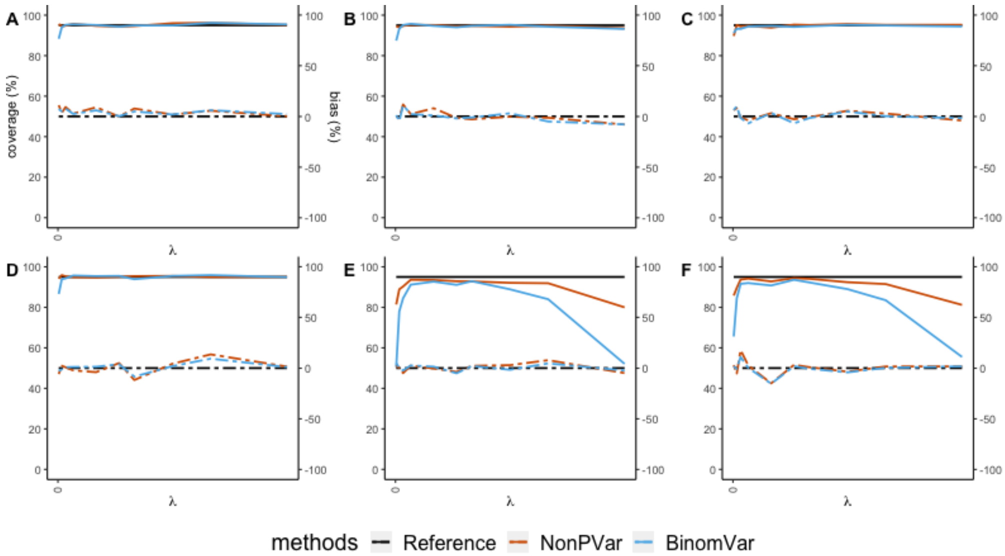

Figure S40: Empirical coverage of the 95% CIs (solid lines, left axis) and relative bias (dashed lines, right axis) for for low mutational load (=1%) in different scenarios, related to Fig. 3. X-axis represents varying concentration of mutant type from low to high. (A) only sampling variation and random partitioning with 3 replicates (B) 3% pipetting error (C) 20% partition loss (D) coefficient of variation of 10% in partition size (E) misclassification with 0.01% false positive rate and 5% false negative rate (F) all variation included. The reference (black solid line) for empirical coverage is set at 95%. The constructed CIs are supposed to cover the true values in 95% of the time. The closer other solid lines are to this reference, the better the CIs are. The reference (black dashed line) is set at 0%. The closer other dashed lines are to this reference, the lower the relative bias is.

4.2 Medium Mutational Load

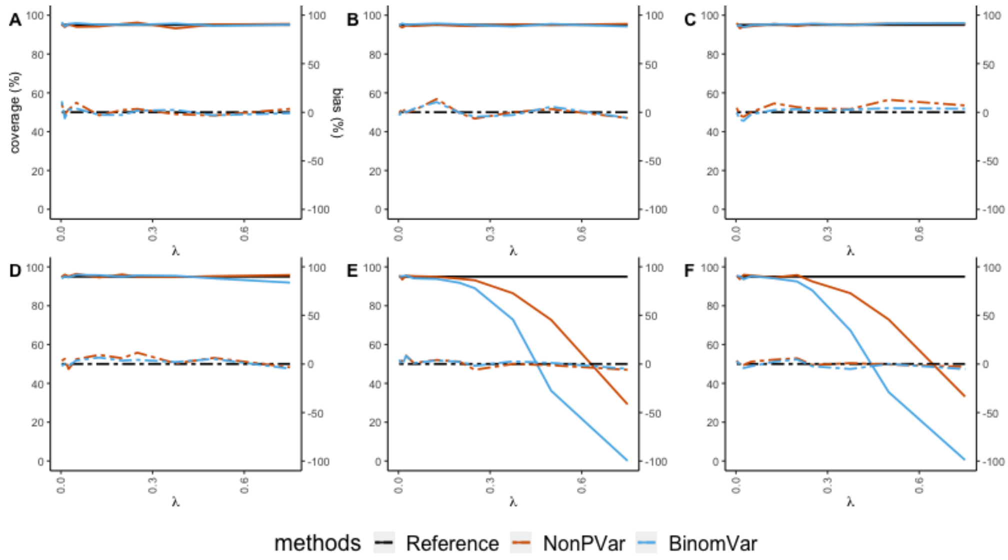

Figure S41: Empirical coverage of the 95% CIs (solid lines, left axis) and relative bias (dashed lines, right axis) for for medium mutational load (=33%) in different scenarios, related to Fig. 3. X-axis represents varying concentration of mutant type from low to high. (A) only sampling variation and random partitioning with 3 replicates (B) 3% pipetting error (C) 20% partition loss (D) coefficient of variation of 10% in partition size (E) misclassification with 0.01% false positive rate and 5% false negative rate (F) all variation included. The reference (black solid line) for empirical coverage is set at 95%. The constructed CIs are supposed to cover the true values in 95% of the time. The closer other solid lines are to this reference, the better the CIs are. The reference (black dashed line) is set at 0%. The closer other dashed lines are to this reference, the lower the relative bias is.

4.3 High Mutational Load

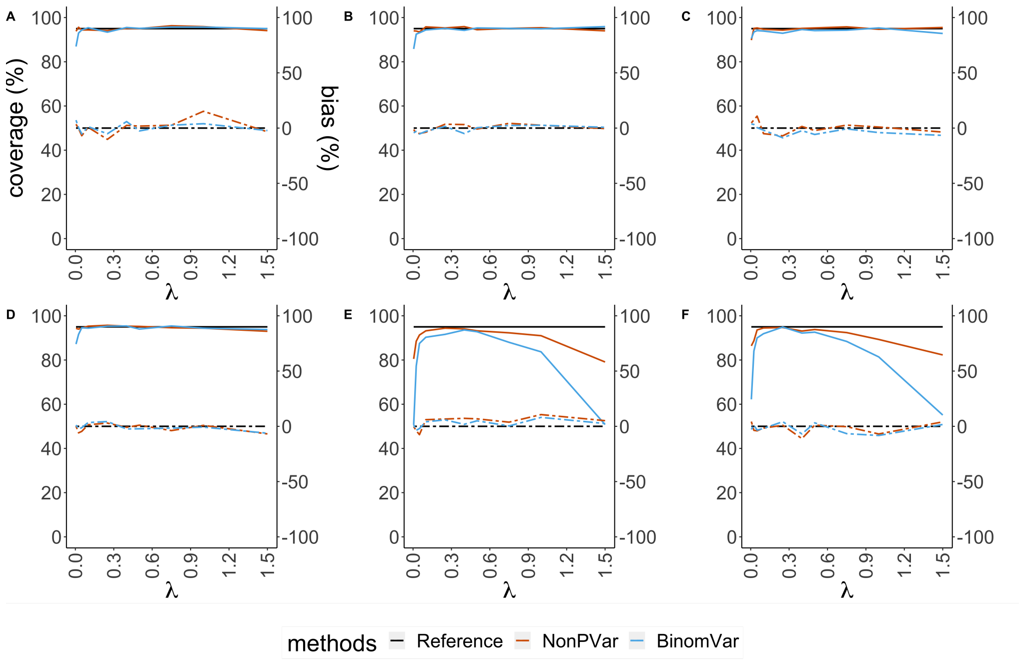

Figure S42: Empirical coverage of the 95% CIs (solid lines, left axis) and relative bias (dashed lines, right axis) for for high mutational load (=99%) in different scenarios, related to Fig. 3. X-axis represents varying concentration of mutant type from low to high. (A) only sampling variation and random partitioning with 3 replicates (B) 3% pipetting error (C) 20% partition loss (D) coefficient of variation of 10% in partition size (E) misclassification with 0.01% false positive rate and 5% false negative rate (F) all variation included. The reference (black solid line) for empirical coverage is set at 95%. The constructed CIs are supposed to cover the true values in 95% of the time. The closer other solid lines are to this reference, the better the CIs are. The reference (black dashed line) is set at 0%. The closer other dashed lines are to this reference, the lower the relative bias is.

5 DNA Shearing Index

5.1 Low DSI

5.1.1 Relative Bias

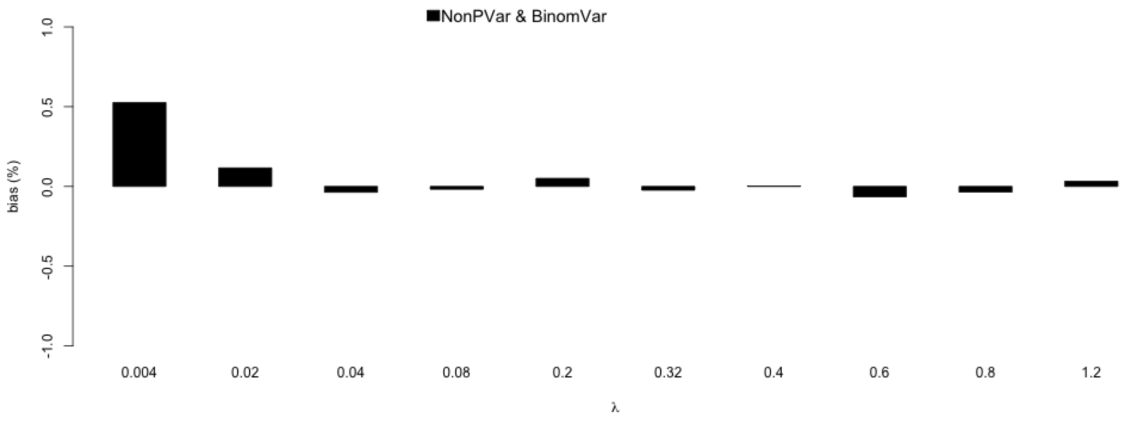

Figure S43: Relative bias of the estimator of dsi in low DSI (=20%, 3 replicates) multiplex with only sampling variation and random partitioning, related to Fig. 2.

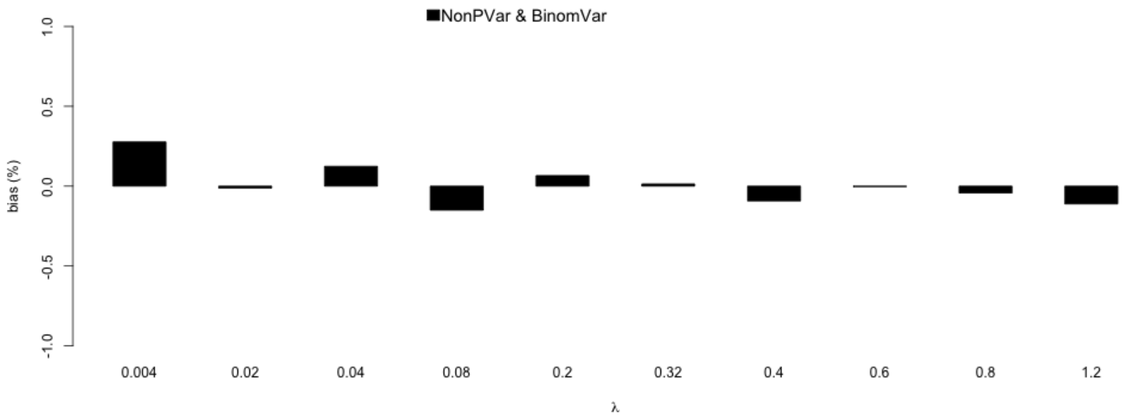

Figure S44: Relative bias of the estimator of dsi in low DSI (=20%, 3 replicates) multiplex with additional pipetting error, related to Fig. 2.

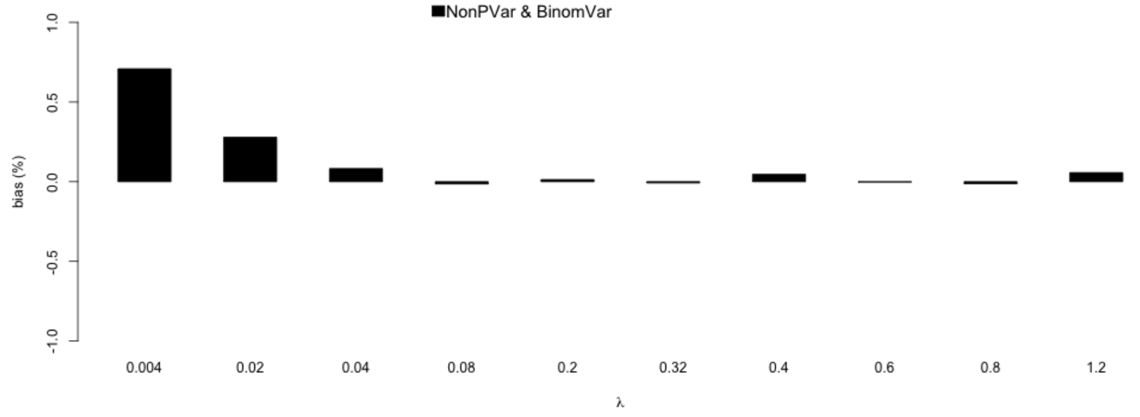

Figure S45: Relative bias of the estimator of dsi in low DSI (=20%, 3 replicates) multiplex with additional partition loss, related to Fig. 2.

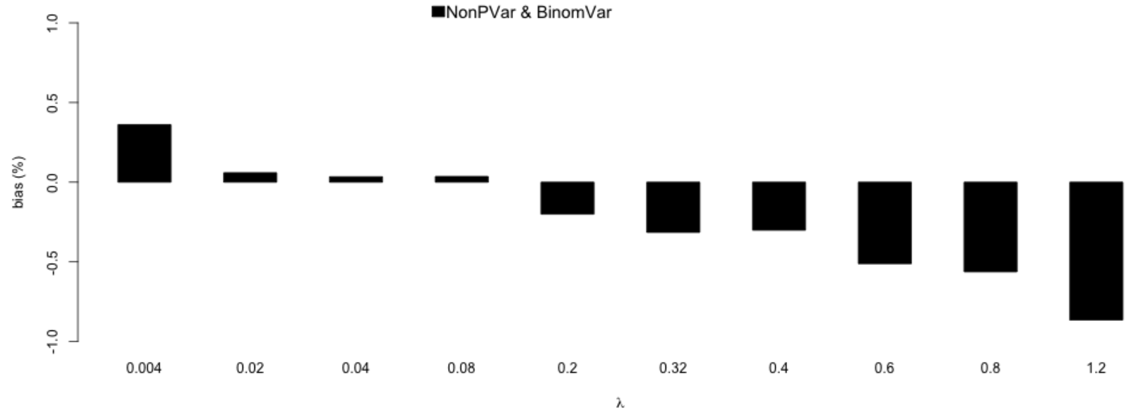

Figure S46: Relative bias of the estimator of dsi in low DSI (=20%, 3 replicates) multiplex with additional partition size variation, related to Fig. 2.

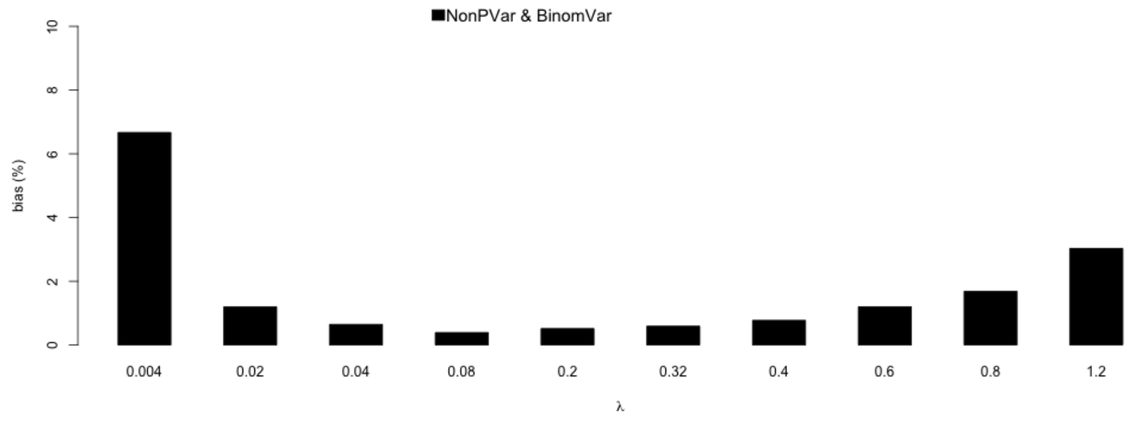

Figure S47: Relative bias of the estimator of dsi in low DSI (=20%, 3 replicates) multiplex with additional misclassification, related to Fig. 2.

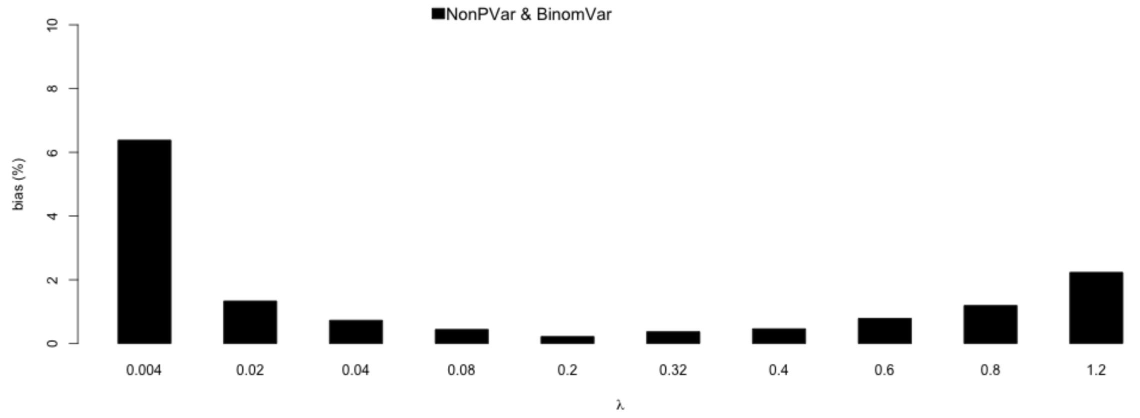

Figure S48: Relative bias of the estimator of dsi in low DSI (=20%, 3 replicates) multiplex with all sources of variation, related to Fig. 2.

5.1.2 Absolute Bias

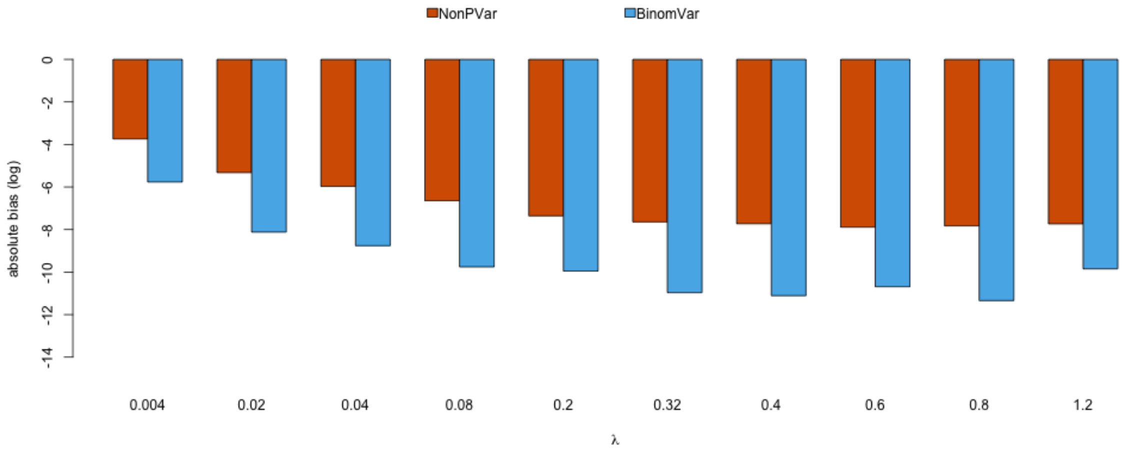

Figure S49: Absolute bias of the variance estimator of dsi in low DSI (=20%, 3 replicates) multiplex with only sampling variation and random partitioning, related to Fig. 2. Note the absolute bias is on the log scale. Smaller on the log scale means closer to 0 on the original scale. Opposite to the relative bias plots, methods with longer bars (extending further down) are better.

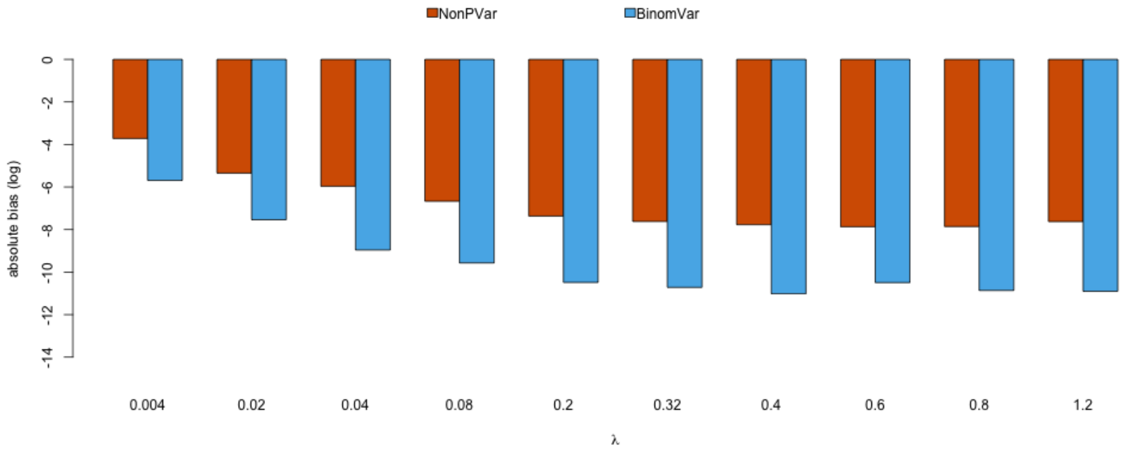

Figure S50: Absolute bias of the variance estimator of dsi in low DSI (=20%, 3 replicates) multiplex with additional pipetting error, related to Fig. 2. Note the absolute bias is on the log scale. Smaller on the log scale means closer to 0 on the original scale. Opposite to the relative bias plots, methods with longer bars (extending further down) are better.

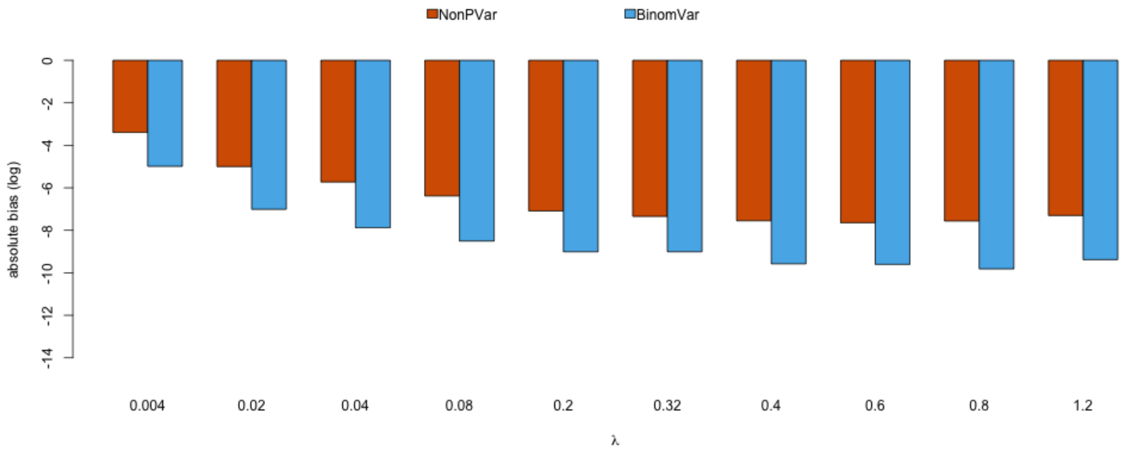

Figure S51: Absolute bias of the variance estimator of dsi in low DSI (=20%, 3 replicates) multiplex with additional partition loss, related to Fig. 2. Note the absolute bias is on the log scale. Smaller on the log scale means closer to 0 on the original scale. Opposite to the relative bias plots, methods with longer bars (extending further down) are better.

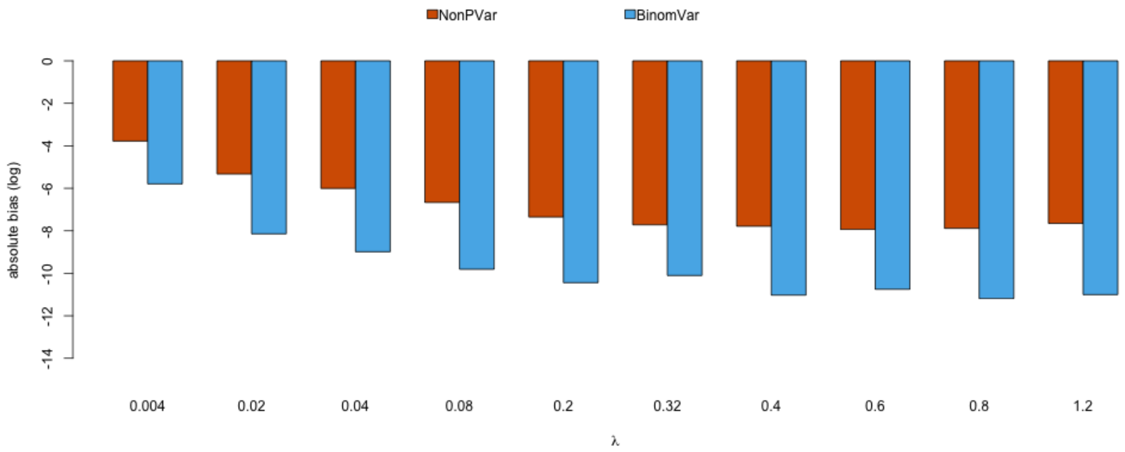

Figure S52: Absolute bias of the variance estimator of dsi in low DSI (=20%, 3 replicates) multiplex with additional partition size variation, related to Fig. 2. Note the absolute bias is on the log scale. Smaller on the log scale means closer to 0 on the original scale. Opposite to the relative bias plots, methods with longer bars (extending further down) are better.

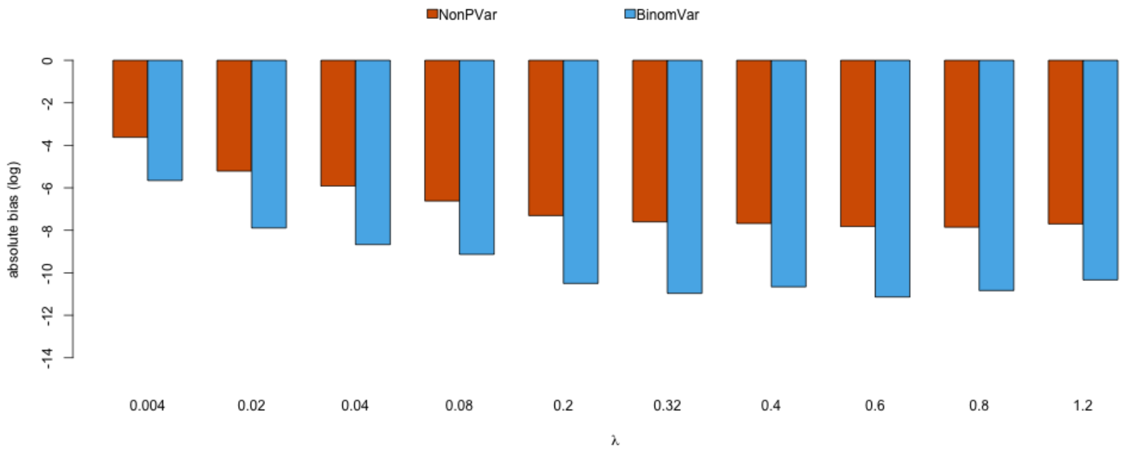

Figure S53: Absolute bias of the variance estimator of dsi in low DSI (=20%, 3 replicates) multiplex with additional misclassification, related to Fig. 2. Note the absolute bias is on the log scale. Smaller on the log scale means closer to 0 on the original scale. Opposite to the relative bias plots, methods with longer bars (extending further down) are better.

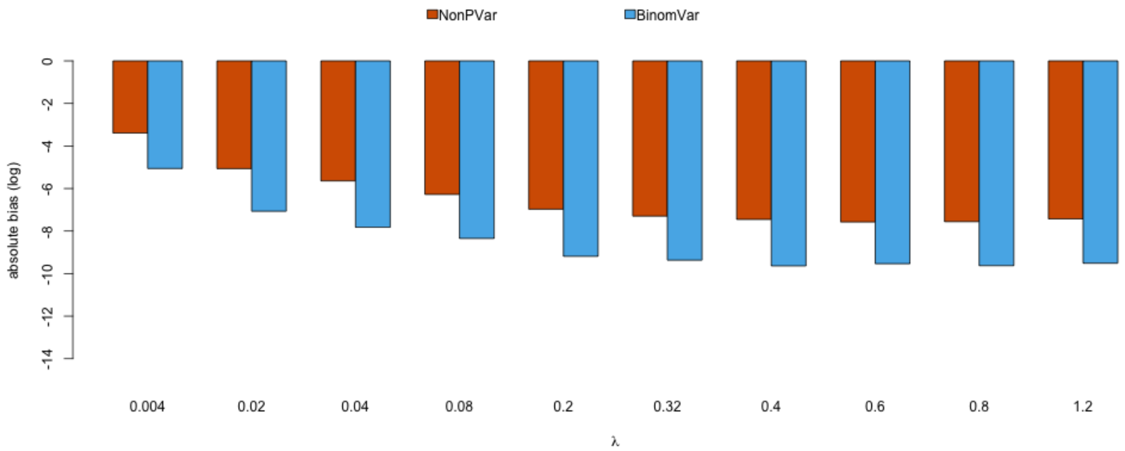

Figure S54: Absolute bias of the variance estimator of dsi in low DSI (=20%, 3 replicates) multiplex with all sources of variation, related to Fig. 2. Note the absolute bias is on the log scale. Smaller on the log scale means closer to 0 on the original scale. Opposite to the relative bias plots, methods with longer bars (extending further down) are better.

5.1.3 Variance Estimates

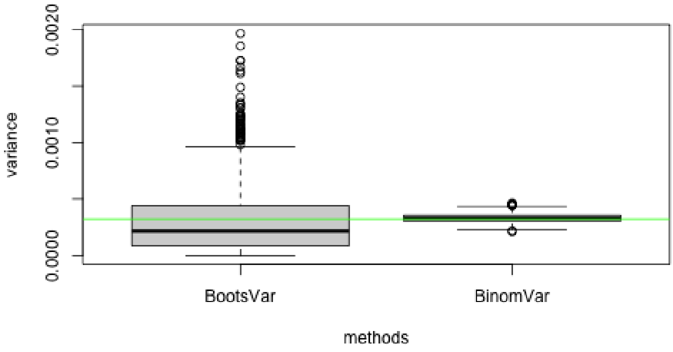

Figure S55: Variance estimates of simulation runs in low DSI (=20%, 3 replicates) multiplex with only sampling variation and random partitioning in low concentration setting of $\lambda_{AB} = 0.004$, related to Fig. 2. The horizontal line is the estimated variance in the simulation (a good approximation to the true variance).

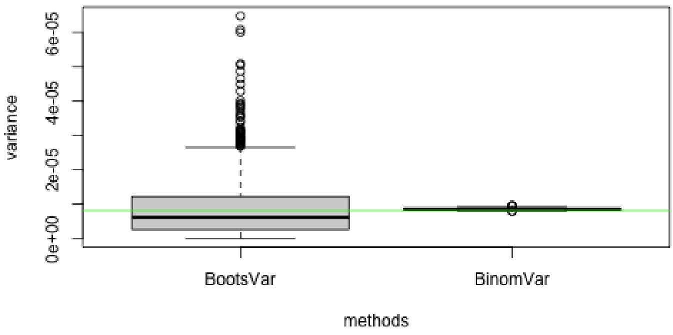

Figure S56: Variance estimates of simulation runs in low DSI (=20%, 3 replicates) multiplex with only sampling variation and random partitioning in medium concentration setting of $\lambda_{AB} = 0.2$, related to Fig. 2. The horizontal line is the estimated variance in the simulation (a good approximation to the true variance).

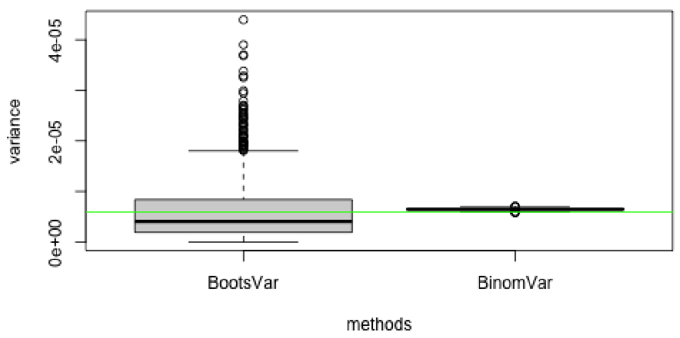

Figure S57: Variance estimates of simulation runs in low DSI (=20%, 3 replicates) multiplex with only sampling variation and random partitioning in high concentration setting of $\lambda_{AB} = 1.2$, related to Fig. 2. The horizontal line is the estimated variance in the simulation (a good approximation to the true variance).

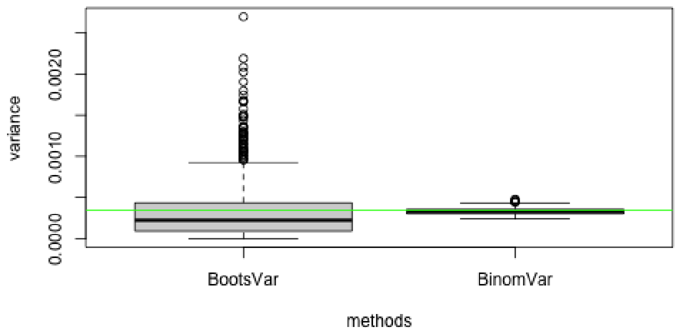

Figure S58: Variance estimates of simulation runs in low DSI (=20%, 3 replicates) multiplex with additional pipetting error in low concentration setting of $\lambda_{AB} = 0.004$, related to Fig. 2. The horizontal line is the estimated variance in the simulation (a good approximation to the true variance).

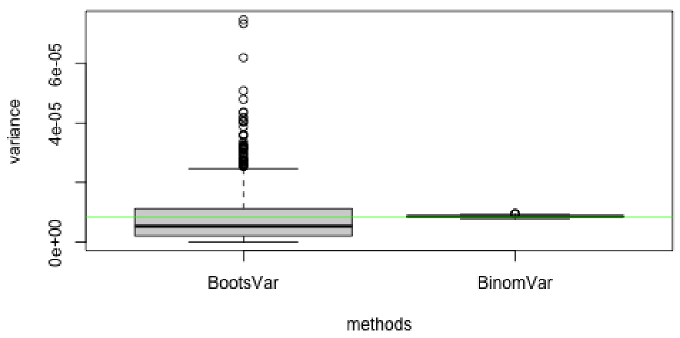

Figure S59: Variance estimates of simulation runs in low DSI (=20%, 3 replicates) multiplex with additional pipetting error in medium concentration setting of $\lambda_{AB} = 0.2$, related to Fig. 2. The horizontal line is the estimated variance in the simulation (a good approximation to the true variance).

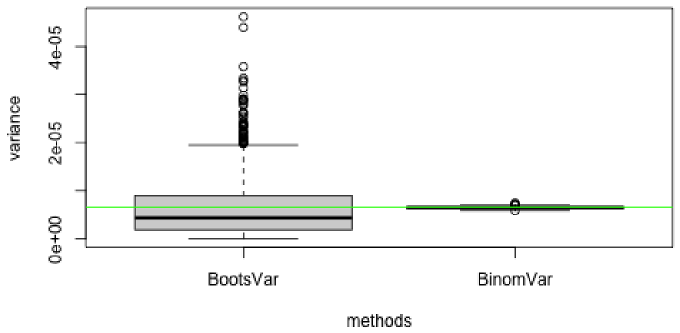

Figure S60: Variance estimates of simulation runs in low DSI (=20%, 3 replicates) multiplex with additional pipetting error in high concentration setting of $\lambda_{AB} = 1.2$, related to Fig. 2. The horizontal line is the estimated variance in the simulation (a good approximation to the true variance).

5.2 Medium DSI

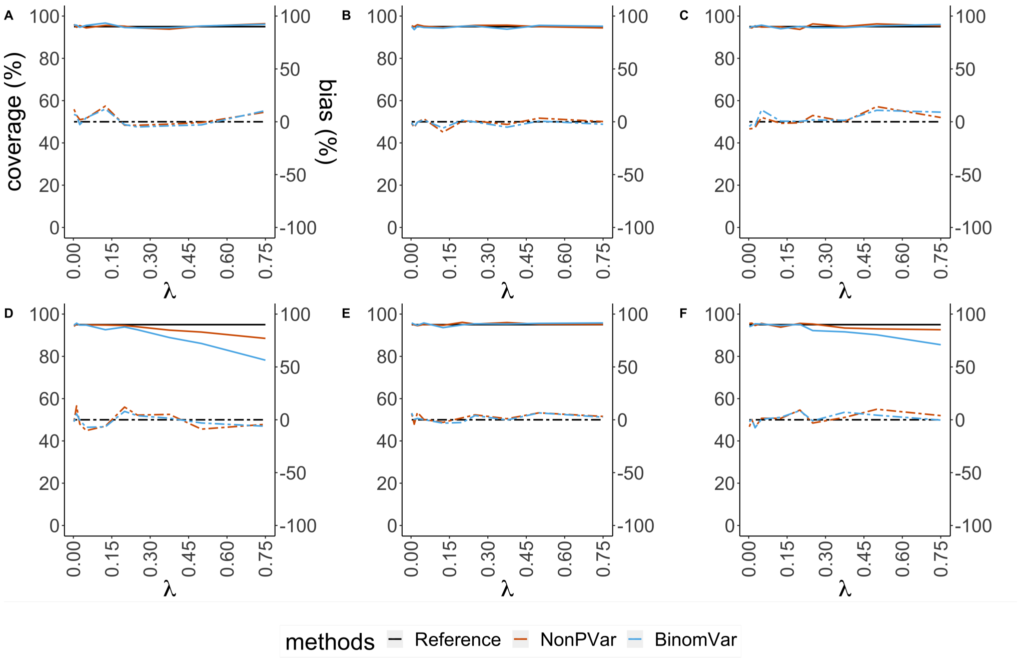

Figure S61: Empirical coverage of the 95% CIs (solid lines, left axis) and relative bias (dashed lines, right axis) for medium DSI (=50%, that is, 50% of the target molecules got sheared) in different scenarios, related to Fig. 2. X-axis represents varying concentration of intact molecules from low to high. (A) only sampling variation and random partitioning with 3 replicates (B) 3% pipetting error (C) 20% partition loss (D) coefficient of variation of 10% in partition size (E) misclassification with 0.01% false positive rate and 5% false negative rate (F) all variation included. The reference (black solid line) for empirical coverage is set at 95%. The constructed CIs are supposed to cover the true values in 95% of the time. The closer other solid lines are to this reference, the better the CIs are. The reference (black dashed line) is set at 0%. The closer other dashed lines are to this reference, the lower the relative bias is.

5.3 High DSI

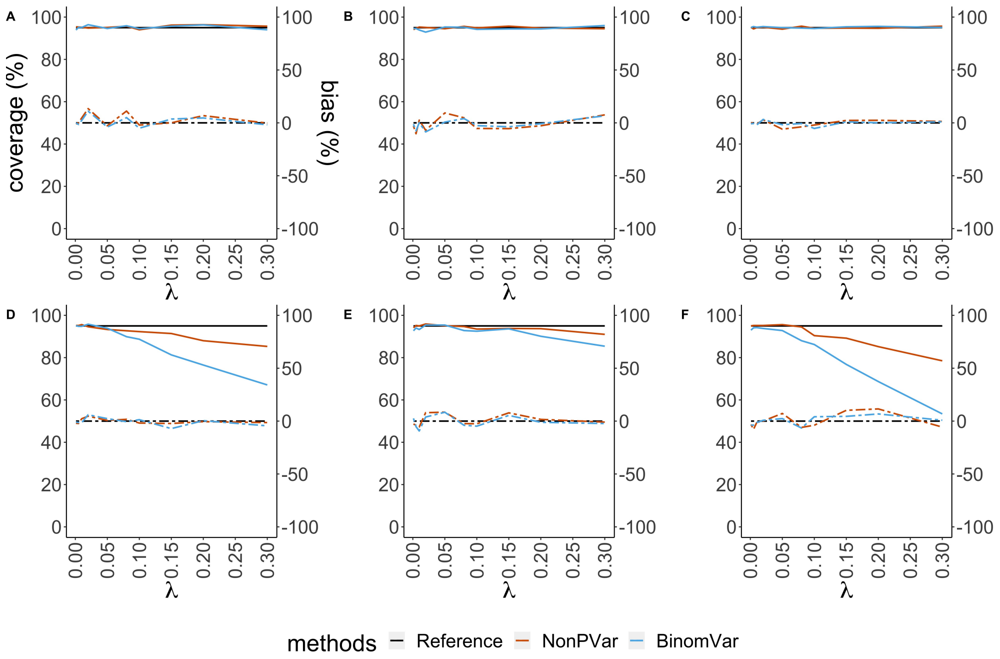

Figure S62: Empirical coverage of the 95% CIs (solid lines, left axis) and relative bias (dashed lines, right axis) for high DSI (=80%, that is, 80% of the target molecules got sheared) in different scenarios, related to Fig. 2. X-axis represents varying concentration of intact molecules from low to high. (A) only sampling variation and random partitioning with 3 replicates (B) 3% pipetting error (C) 20% partition loss (D) coefficient of variation of 10% in partition size (E) misclassification with 0.01% false positive rate and 5% false negative rate (F) all variation included. The reference (black solid line) for empirical coverage is set at 95%. The constructed CIs are supposed to cover the true values in 95% of the time. The closer other solid lines are to this reference, the better the CIs are. The reference (black dashed line) is set at 0%. The closer other dashed lines are to this reference, the lower the relative bias is.

6 Empirical data Analysis

Methods	Runtime (s)
**NonPVar**	0.0065
**BinomVar**	0.0148
**GLMM**	13.4445
**Delta Method**	0.0066

Table S1: Computation time (4GB 1600 MHz DDR3) for 1 marker with 80% of partitions positive and 3 replicates, related to STAR Methods. For BinomVar, the bootstrap iteration is set at 1000.

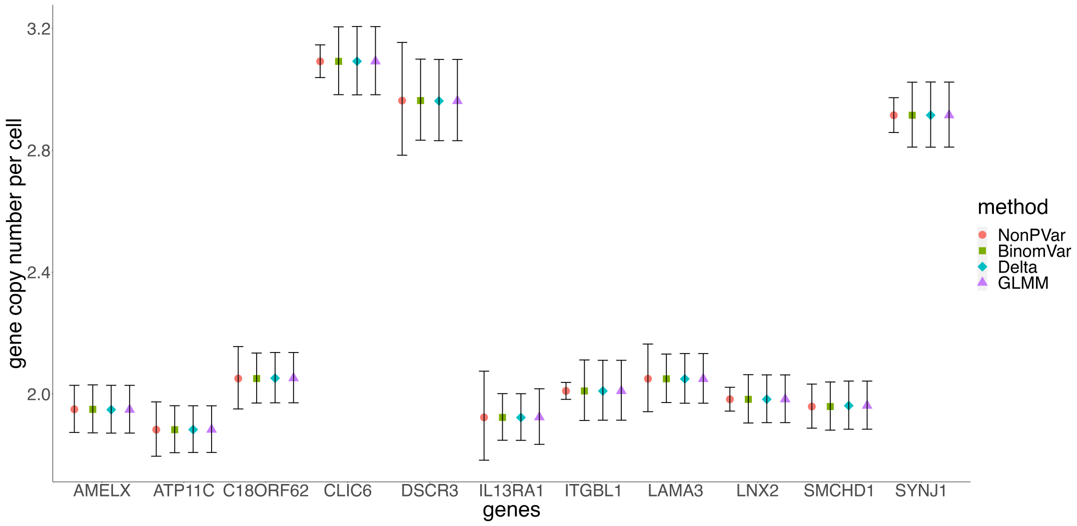

Figure S63: Copy numbers in sample 6 after normalization using the RPP30 locus (accounting for inter-replicate variability), data are represented as mean +/- SEM, related to Fig. 4.
